# Engineering the Immune Response to Biomaterials

**DOI:** 10.1002/advs.202414724

**Published:** 2025-04-15

**Authors:** Abolfazl Salehi Moghaddam, Mehran Bahrami, Einollah Sarikhani, Rumeysa Tutar, Yavuz Nuri Ertas, Faleh Tamimi, Ali Hedayatnia, Clotilde Jugie, Houman Savoji, Asma Talib Qureshi, Muhammad Rizwan, Chima V. Maduka, Nureddin Ashammakhi

**Affiliations:** ^1^ Department of Bioengineering P.C. Rossin College of Engineering & Applied Science Lehigh University Bethlehem PA 18015 USA; ^2^ Department of Mechanical Engineering & Mechanics Lehigh University Bethlehem PA 18015 USA; ^3^ Department of Nano and Chemical Engineering University of California San Diego La Jolla CA 92093 USA; ^4^ Department of Chemistry Faculty of Engineering, Istanbul University‐Cerrahpaşa Istanbul, Avcılar 34320 Turkey; ^5^ Department of Biomedical Engineering Erciyes University Kayseri 38039 Turkey; ^6^ ERNAM – Nanotechnology Research and Application Center Erciyes University Kayseri 38039 Turkey; ^7^ College of Dental Medicine Qatar University Health Qatar University P.O. Box 2713 Doha Qatar; ^8^ Azrieli Research Center Centre Hospitalier Universitaire Sainte‐Justine Montreal QC H3T 1C5 Canada; ^9^ Institute of Biomedical Engineering, Department of Pharmacology and Physiology Faculty of Medicine Montreal Quebec H3T 1J4 Canada; ^10^ Montreal TransMedTech Institute iTMT Montreal Quebec H3T 1J4 Canada; ^11^ Department of Biomedical Engineering Michigan Technological University Houghton MI 49931 USA; ^12^ Health Research Institute Michigan Technological University Houghton MI 49931 USA; ^13^ BioFrontiers Institute University of Colorado Boulder CO 80303 USA; ^14^ Institute for Quantitative Health Science and Engineering (IQ) and Department of Biomedical Engineering (BME) Colleges of Engineering and Human Medicine Michigan State University East Lansing MI 48824 USA; ^15^ Department of Bioengineering Samueli School of Engineering University of California Los Angeles Los Angeles CA 90095 USA

**Keywords:** biomaterial, bioactive, biodegradable, immune reaction modulation, inflammation

## Abstract

Biomaterials are increasingly used as implants in the body, but they often elicit tissue reactions due to the immune system recognizing them as foreign bodies. These reactions typically involve the activation of innate immunity and the initiation of an inflammatory response, which can persist as chronic inflammation, causing implant failure. To reduce these risks, various strategies have been developed to modify the material composition, surface characteristics, or mechanical properties of biomaterials. Moreover, bioactive materials have emerged as a new class of biomaterials that can induce desirable tissue responses and form a strong bond between the implant and the host tissue. In recent years, different immunomodulatory strategies have been incorporated into biomaterials as drug delivery systems. Furthermore, more advanced molecule and cell‐based immunomodulators have been developed and integrated with biomaterials. These emerging strategies will enable better control of the immune response to biomaterials and improve the function and longevity of implants and, ultimately, the outcome of biomaterial‐based therapies.

## Graphical abstract

This paper reviews immune responses to biomaterials, stages of evolution in relation to managing these responses ranging from preventing to modulating them. It also discusses state‐of‐the‐art strategies to modulate these responses and modes of their delivery. The paper opens up new directions for future research toward developing smart implants that can autonomously function and self‐repair in our bodies.

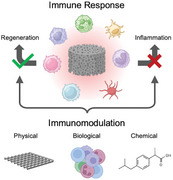

## 1 Introduction

There is a global rise in life expectancy and in aging population.^[^ ^1^ ^]^ Aging is associated with increased risk of trauma, tissue injury, and organ failure, which necessitate treatment using biomaterials and transplants to restore or replace injured tissues and failed organs. As the body ages, the immune system undergoes significant changes, often referred to as immunosenescence.^[^ ^2^ ^]^ Immunosenescence includes a decline in the function of both the innate and adaptive immune systems.^[^ ^2^, ^3^ ^]^ These changes increase susceptibility to infections and lead to impaired wound healing.^[^ ^4^ ^]^ Consequently, aging individuals exhibit a heightened incidence of chronic inflammatory conditions, often termed as “inflammaging”.^[^ ^5^ ^]^ This aging‐related alteration in immune response can impact both the initial innate nonspecific immune reaction and the subsequent chronic inflammation regarding biomaterials.^[^ ^5^ ^]^ There is a growing reliance on biomaterials and organ transplants to address these age‐related health challenges. However, biomaterials trigger immune reactions,^[^ ^6^ ^]^ which can negatively impact the function of the implanted biomaterials and health of the patient.^[^ ^7^ ^]^ These reactions include an innate nonspecific immune response, that leads inflammation.^[^ ^8^ ^]^ The initial inflammatory response is usually limited, but may become chronic^[^ ^9^ ^]^ and cause problems.^[^ ^10^ ^]^

During the immune response to biomaterials, pro‐inflammatory macrophages (MPs) release various molecules that amplify the immune response.^[^ ^10^ ^]^ These inflammatory mediators can disrupt tissue homeostasis, leading to issues such as bone resorption and implant loosening, particularly evident in bone implants,^[^ ^11^ ^]^ where the activation of osteoclasts occurs.^[^ ^12^ ^]^ Chronic inflammation may also result in fibrous tissue formation,^[^ ^11^ ^]^ which may impair the function of implanted devices,^[^ ^13^ ^]^ such as those that may occur with glucose sensors.^[^ ^14^, ^15^, ^16^ ^]^ These complications highlight the critical need for biomaterials that can seamlessly integrate with host tissues without eliciting adverse immune responses.

Initially, efforts to mitigate immune reactions focused on developing bioinert materials that minimize interaction with the body's immune system, leading to the development of the first successful (Charnley) femoral head prosthesis using Vitallium (a cobalt‐based alloy) stainless steel.^[^ ^17^ ^]^ Although useful, bioinert materials lack the integration with surrounding tissues, which limits implant durability.^[^ ^18^, ^19^ ^]^ Therefore, the introduction of bioactive materials, such as bioactive glass (BaG) and other ceramic biomaterials,^[^ ^20^ ^]^ led to a shift in focus to biomaterial “bioactivity”, in which biomaterials can induce reactions that enhance integration with the surrounding host tissue.^[^ ^21^ ^]^ Bioactive materials offer the advantage of tailored biodegradation rates, allowing customization based on specific medical applications.^[^ ^22^, ^23^ ^]^ However, the inherent rigidity of glass materials led researchers to explore biodegradable polymers, which degrade more flexibly but can result in the formation of fibrous tissue alongside the degraded biomaterial.^[^ ^24^, ^25^ ^]^ This observation prompted innovative approaches in tissue engineering, particularly for small joint repairs, by harnessing the body's tissue response to support in situ tissue engineering.^[^ ^26^ ^]^

Despite these advancements, the chronic inflammatory response associated with the degradation of biodegradable materials poses significant challenges, including implant extrusion.^[^ ^27^ ^]^ To address these issues, research has increasingly focused on controlling inflammatory reactions through various strategies.^[^ ^28^, ^29^ ^]^ One primary approach involves the development of next generation of biomaterials where the focus is on preventing unwanted immune (inflammatory) reactions. To achieve this, various chemical strategies were explored. These include the use of agents such as steroids^[^ ^30^, ^31^ ^]^ or nonsteroidal anti‐inflammatory drugs (NSAIDs)^[^ ^32^, ^33^, ^34^, ^35^, ^36^ ^]^ to reduce fibrosis,^[^ ^37^ ^]^ or anti‐osteolytic drugs^[^ ^38^ ^]^ to prevent bone resorption, or anti‐proliferative agents^[^ ^39^, ^40^, ^41^ ^]^ to prevent restenosis of blood vessels^[^ ^40^, ^42^ ^]^ following vascular stent implantation. However, these drugs are not specific and suffer from the risk of causing unwanted side effects.

More recently, new strategies for the modification of immune responses have been explored, including the use of specific tissue response modulating agents, such as agents targeting specifically colony stimulating factor 1 receptor,^[^ ^43^ ^]^ that regulates MP differentiation and activation.^[^ ^44^ ^]^ These agents enable a more selective and effective way of modifying the immune response by altering the MP phenotype from pro‐inflammatory to anti‐inflammatory states,^[^ ^45^ ^]^ and enhancing healing by regeneration^[^ ^46^, ^47^, ^48^ ^]^ instead of fibrosis.^[^ ^49^ ^]^ Additionally, genetic approaches using small interfering RNA (siRNA) and microRNA (miRNA) have also been studied to precisely target and regulate genes or pathways involved in inflammation, offering a more refined method of controlling immune responses to biomaterials.^[^ ^50^ ^]^

Physical strategies have also been employed in modulating immune responses by altering the surface properties of biomaterials. Modifications in surface roughness, topography, chemistry, and charge can significantly influence the interactions between the implant and the biological environment.^[^ ^51^, ^52^, ^53^ ^]^ Modifying implant surface patterns can improve implant biocompatibility and enhance its integration.^[^ ^54^ ^]^ For example, nanopatterning (spacing, spikes, arrays, orientation, and size) was found to modulate biocompatibility.^[^ ^55^ ^]^ Surface modification can promote specific interactions with surrounding tissues, thereby improving implant integration and reducing adverse immune responses.^[^ ^56^, ^57^ ^]^ By reducing inflammation, fibrosis, and foreign body reaction (FBR),^[^ ^58^, ^59^ ^]^ the success of implants can be enhanced.^[^ ^60^ ^]^ This strategy has also been explored for modulating the immune system for certain therapeutic purposes, such as stimulating antigen presentation,^[^ ^61^, ^62^ ^]^ activating immune cells,^[^ ^63^ ^]^ or inducing immune tolerance to improve transplant survival.^[^ ^64^ ^]^

Adjusting the mechanical properties of biomaterials is another strategy that has been investigated to achieve desired interactions with the biological environment by enhancing antigen presentation or stimulating immune cells.^[^ ^54^, ^57^, ^65^, ^66^, ^67^, ^68^ ^]^ For instance, stiffness and elasticity of biomaterials can influence the immune response, as they can affect the adhesion, migration, activation, and polarization of immune cells.^[^ ^57^ ^]^ For optimal outcome, implant mechanical properties should match those of the target tissue, such as the brain,^[^ ^69^ ^]^ where it was found that softer materials lead to reduced inflammatory reaction.^[^ ^70^ ^]^ Moreover, the development of stimuli‐responsive biomaterials that can adapt their properties in response to environmental changes, such as pH and temperature shifts, represents a cutting‐edge approach in immunomodulation.^[^ ^71^ ^]^ These smart materials can potentially be used for modulating immune response, in applications such as vaccination and cancer immunotherapy,^[^ ^72^, ^73^ ^]^ where precise delivery and release of immunomodulatory agents are crucial for achieving desired response.

Biological strategies have also been explored for immunomodulation, leveraging the capabilities of cells like stem cells, MPs, and dendritic cells (DCs) to release anti‐inflammatory cytokines,^[^ ^74^ ^]^ promote tissue regeneration,^[^ ^75^ ^]^ and induce immune tolerance.^[^ ^76^ ^]^ In addition, cell derived products such as extracellular vesicles (EVs)^[^ ^77^, ^78^, ^79^, ^80^, ^81^, ^82^, ^83^ ^]^ and cell‐derived membranes^[^ ^84^, ^85^ ^]^ have potential for use in modulating immune response and enhancing tissue regeneration, making them valuable tools in the development of next‐generation biomaterials.

Combined strategies which integrate physical, chemical, and biological approaches can be used for achieving synergistic enhancement of immunomodulation. For example, co‐delivery of small molecule inhibitors with cells,^[^ ^86^ ^]^ the incorporation of physical cues with cell‐based therapies,^[^ ^87^ ^]^ or the creation of materials that combine multiple functional properties, such as surface texture with controlled release of bioactive molecules can significantly improve regenerative outcomes.^[^ ^88^ ^]^ The primary goal of combined strategies is to integrate the advantages of individual approaches.^[^ ^54^ ^]^ This multi‐modal approach is already being tested in animals to evaluate its effectiveness.^[^ ^54^ ^]^

**Figure 1:**
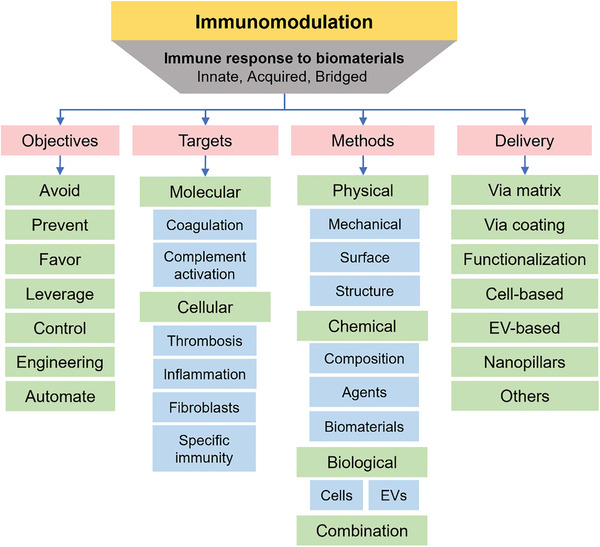
Immune response to biomaterials and its modulation, including objectives, targets, methods, and modes of delivery.

Overall, the aim of research on immunomodulation has evolved from merely avoiding adverse tissue reactions to actively preventing and controlling them. It is also becoming possible to design biomaterials that induce predesigned tissue reaction. Advances in biomaterial science now allow for the design of smart implants that can autonomously respond to their microenvironment, mimicking the dynamic and self‐healing properties of natural tissues.^[^ ^89^ ^]^ Accordingly, an important aspect of such biomaterials is also their capability to induce desired and appropriate immune response. To gain a thorough understanding of the current state‐of‐the‐field and provide insight for next steps, this review aims to link dots, synthesize information into key concepts, and analyze research and development directions. This will provide scientists and clinicians with lessons learnt, and current ideas, aiding the development of innovative solutions that can shape the future of implant design (**Figure** **1**).

## 2 Immune Response to Biomaterials

**Figure 2:**
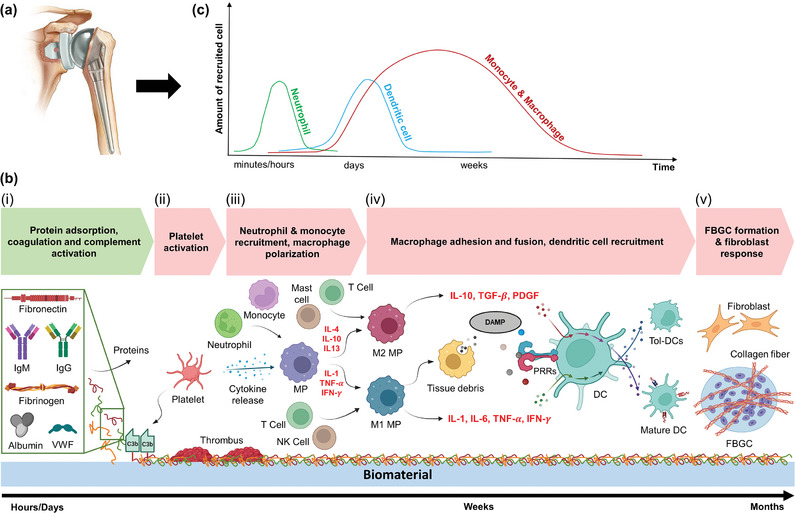
Illustration showing a shoulder prosthesis (a). b) Molecular events (green box) and cellular responses (pink boxes) to implanted biomaterial. Molecular events include: (i) the non‐specific proteins (IgM: Immunoglobulin M, IgG: Immunoglobulin G, VWF:  von Willebrand factor) attachment to the surface of the biomaterial, and the activation of coagulation and complement system. Cellular responses include (ii) platelet, (iii) neutrophil and monocyte recruitment, (iv) macrophage (MP) adhesion and dendritic cell (DC) recruitment, and (v) foreign body giant cell (FBGC) formation and fibroblast response. c) Non‐specific cellular immune response to implanted biomaterials over time. (C3b: larger element of complement component, TGF‐β: transforming growth factor beta, TNF‐α: tumor necrosis factor alpha, PRR: Pathogen recognition receptors, IL: interleukin, IFN‐γ: interferon‐gamma, DAMP: damage‐associated molecular pattern, PDGF: platelet‐derived growth factor). Adapted with permission.^[^ ^100^ ^]^ Licensed under Creative Commons Attribution (CC BY), created with BioRender.com.

In the body, biomaterials elicit an immune response,^[^ ^90^ ^]^ which can be classified into acute and chronic reactions, as well as nonspecific and specific responses.^[^ ^91^, ^92^ ^]^ Acute immune response takes place within minutes to hours after implantation,^[^ ^93^ ^]^ and it usually subsides within a few days. It primarily involves nonspecific mechanisms that inlude the attraction of immune cells such as neutrophils and MPs to the implant site.^[^ ^94^, ^95^ ^]^ On the other hand, chronic immune response develops weeks to months after implantation.^[^ ^96^, ^97^, ^98^ ^]^ It involves both nonspecific and specific mechanisms, including the activation of MPs and T cells, and leads to fibrous tissue encapsulation of the implant,^[^ ^96^, ^99^ ^]^ which may interfere with the implant function and causes long‐term problems.^[^ ^93^ ^]^ Moreover, chronic inflammatory response can lead to tissue damage and implant failure.^[^ ^12^ ^]^ These immune responses include both molecular and cellular mechanisms, details of which are discussed in the following subsections (**Figure** **2**). The objective of this section is to provide a background of these immune responses that are useful for understanding the developments in engineered immune responses that follow in the next sections of this paper.

### 2.1 Non‐Specific Immunity

**Figure 3:**
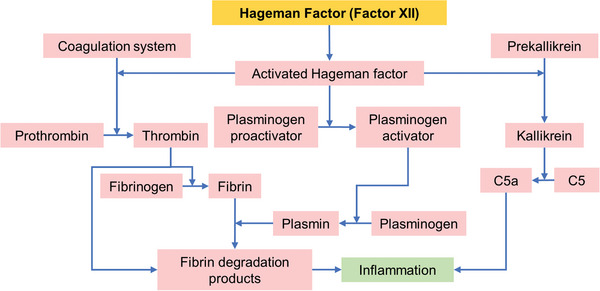
Illustration showing the interaction between various molecular systems that include the activation of Hageman factor (FXII) by collagen following injury, which leads to the activation of coagulation, fibrinolytic, and complement systems. At the end, the molecular reaction leads to inflammatory cellular response. (C5: complement component 5, C5a: smaller element of complement component 5). Adapted with permission.^[^ ^105^ ^]^ 2019, CRC Press Imprint, Taylor & Francis Group.

**Figure 4:**
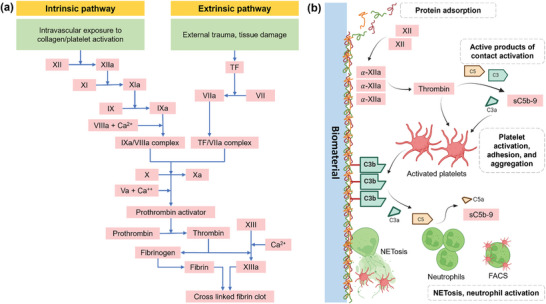
Schematic showing: a) the components and activation of coagulation system through intrinsic or extrinsic pathways resulting into cross linked fibrin clot.^[^ ^106^ ^]^ b) interaction of the coagulation system with complement and platelet response including platelet activation, adhesion, and aggregation. The result of this interaction will be neutrophil extracellular trap formation (NETosis), neutrophil activation, and Fluorescence‐activated cell sorting (FACS).^[^ ^107^ ^]^ (Factor XI: amplifies intrinsic pathway; activates factor IX, Factor XIa: active form; converts factor IX to IXa, Factor XII (Hageman factor): initiates intrinsic pathway via contact activation, Factor XIIa: activates factor XI and kallikrein; links coagulation and inflammation, Factor IX: intrinsic pathway; activated by factor XIa, Factor IXa: combines with factor VIIIa to activate factor X, Factor VIII: cofactor for factor IX in intrinsic pathway, Factor VIIIa: activated cofactor; enhances factor IXa activity, Factor X: common pathway; activated by intrinsic and extrinsic pathways, Factor Xa: converts prothrombin to thrombin, Factor V: cofactor in prothrombinase complex (factor Xa activation), Factor Va: active cofactor; accelerates thrombin production, Prothrombin (Factor II): precursor of thrombin, Thrombin (Factor IIa): central enzyme; converts fibrinogen to fibrin, Fibrinogen (Factor I): precursor of fibrin; forms the clot structure, factor XIII: stabilizes fibrin clot via cross‐linking, Factor XIIIa: active form; reinforces clot stability, Factor VII: initiates extrinsic pathway; activates factor X, Factor VIIa: active form; works with tissue factor to activate factor X, TF: tissue factor, Ca2+: Serum calcium, C3: complement component 3, C5: complement component 5, sC5b‐9: soluble complement 5b‐9) Created with BioRender.com.

Immediately within seconds of exposure to blood, the surface of the biomaterial adsorbs different plasma proteins resulting in activated coagulation and innate immunity.^[^ ^101^ ^]^ These proteins include fibrinogen, high‐molecular‐weight kininogen (HK), coagulation factor XI (FXI) and FXII/XIIa, plasma prekallikrein (PK), immunoglobulins (IgG, IgM), complement proteins, albumin, vitronectin, fibronectin, apolipoproteins, and von Willebrand factor (VWF)^[^ ^101^ ^]^ (**Figure** **3**). The type of adsorbed plasma proteins to biomaterials may vary over time with adsorption and desorption, depending on the biomaterial surface hydrophobicity, topography, and charge.^[^ ^102^, ^103^ ^]^ Unlike hydrophilic surfaces, hydrophobic surfaces tend to adsorb more proteins. Consequently, factor XII (Hageman factor) is activated, which begins a chain of reactions that leads to blood clotting (**Figure** **4**). PK can also be turned on by factor XII and help to activate the complement system by breaking down two other proteins, C3 and C5. These interactions between biomaterials, blood clotting, and complement system can cause blood clotting and inflammation, which can affect how the biomaterial will function in the body.^[^ ^104^ ^]^

#### 2.1.1 Molecular Events

##### Coagulation

The activation of coagulation system occurs after the implantation of a biomaterial.^[^ ^101^, ^108^ ^]^ Coagulation system is activated through either intrinsic (tissue factor) or extrinsic (contact activation) pathway^[^ ^109^ ^]^ (Figure 4a). Coagulation can amplify inflammation by activating complement and generating pro‐inflammatory cytokines. Inflammation can further promote coagulation by upregulating tissue factor expression, activating platelets, and impairing fibrinolysis.^[^ ^110^ ^]^ Therefore, coagulation and inflammation form a vicious cycle that can compromise the function of the biomaterial.^[^ ^101^, ^108^, ^111^ ^]^ First, fibrin deposition occurs, followed by the infiltration of neutrophils,^[^ ^95^ ^]^ then MPs^[^ ^112^ ^]^ and later the formation of giant cells and fibrous tissue capsule^[^ ^113^ ^]^ (Figure 2c).

##### Complement System

**Figure 5:**
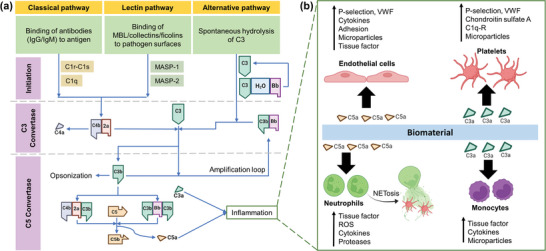
Schematic showing: a) Components of the complement system and its activation through classic, lectin, or alternative pathways.^[^ ^117^ ^]^ Created with BioRender.com. b) Its effect on various cells and the increase in different factors. (C3a: smaller element of complement component 3, C3b: larger element of complement component 3, MASP: mannose‐associated serine protease, ROS: reactive oxygen species, VWF:  von Willebrand factor, MBL: mannose‐binding lectin).^[^ ^101^ ^]^ Created with BioRender.com.

The complement system is a critical component of the innate immune response, comprising over 40 proteins. By activation of the complement system, these proteins work in a highly regulated cascade to recognize foreign substances, such as pathogens or biomaterials, while promoting the adaptive immune reaction and recruiting inflammatory cells.^[^ ^114^ ^]^ When an implant is in contact with blood, complement factors accumulate on its surface and bind to conformationally altered adsorbed proteins, as well as to the implant surface itself.^[^ ^101^, ^107^ ^]^ The complement proteins C3a and C5a can recruit and activate immune cells, such as MPs, neutrophils, and mast cells. The complement cascade is regulated by various soluble and membrane‐bound factors that can inhibit or enhance its activation. The complement system is activated through either classical, lectin, or alternative pathways, which are initiated by molecular patterns or functional groups displayed on the surface of a biomaterial when it is exposed to blood (**Figure** **5**a).^[^ ^115^ ^]^ Healthy host cells are protected against damage by complement system by membrane‐anchored and soluble negative regulators.^[^ ^116^ ^]^ However, implanted biomaterials or transplanted cells usually lack this intrinsic protection against complement activation.

Biomaterial surface can trigger complement activation. Biomaterial surface nucleophiles (−OH or −NH_2_) can bind C3(H_2_O) and reactive C3b.^[^ ^101^, ^116^ ^]^ Surface‐functionalized polymers [e.g., polyethylene glycol (PEGs), poloxamers], hydration‐altered surfaces, and modified adsorbed proteins may result in the formation of C3a and C5a via lectin pathway.^[^ ^101^ ^]^ Titanium (Ti) implants have a porous structure that can adsorb proteins and activate the complement system.^[^ ^118^ ^]^ The activation of the complement system is prothrombotic and pro‐inflammatory. It promotes thrombin generation, adherence of platelets, platelet activation, clot formation, and neutrophil extracellular traps (NETs.^[^ ^119^ ^]^ In addition, it provokes the secretion of VWF and P‐selectin from platelets, triggering and sustaining platelet adhesion and aggregation and the recruitment of leukocytes such as monocytes/MPs.^[^ ^101^ ^]^ To regulate the process, endothelial cells synthesize the complement regulatory factor H (FH), which inhibits the activation of complement by binding to VWF. By simultaneously secreting VWF and FH, activated ECs may enhance platelet adhesion to wounds to ensure healing, while dampening complement's pro‐inflammatory effect to limit tissue damage to bystanders.^[^ ^120^ ^]^

##### Inflammasome

The inflammasome is a multiprotein complex that mediates the production of pro‐inflammatory cytokines such as interleukin‐1β (IL‐1β) and IL‐18, which regulate the immune response.^[^ ^121^ ^]^ It assembles upon recognition of danger signals, which are released following tissue injury caused by implant installation.^[^ ^122^ ^]^ The inflammasome plays a role in various inflammatory processes, ranging from pathogen clearance to chronic inflammation, tissue repair,^[^ ^123^ ^]^ and fibrosis.^[^ ^124^ ^]^ The activation of the NLRP3 type of inflammasome by biomaterials results in the production and secretion of IL‐1β and IL‐18, which amplify the immune reaction and induce fibrosis.^[^ ^123^, ^125^ ^]^

#### 2.1.2 Cellular Responses

##### Platelet Response

**Figure 6:**
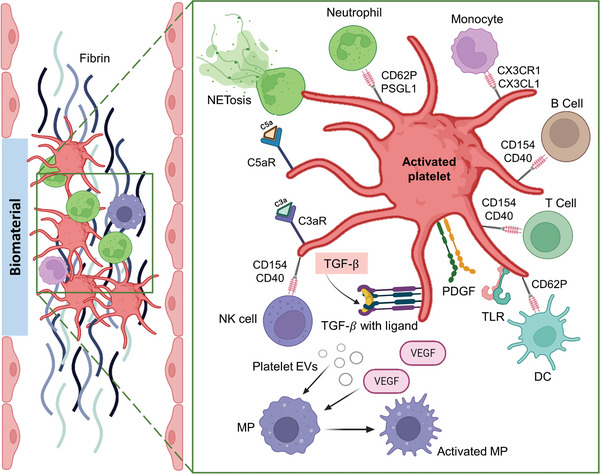
Schematic showing platelet activation as a response to implanted biomaterial, interaction with other types of cells and molecules such as dendritic cell (DC), T‐ and B‐cells, natural killer (NK) cell, monocyte, neutrophil, toll‐like receptors (TLR), platelet‐derived growth factor (PDGF), and transforming growth factor beta (TGF‐β). The receptors for each type of cell are shown as well. Platelet activation is followed by macrophage (MP) activation through released platelet‐extracellular vesicles (EVs) and Vascular endothelial growth factor (VEGF). (CX3CR1: C‐X3‐C motif chemokine receptor 1, CX3CL1: C‐X3‐C chemokine ligand 1, CD40: cluster of differentiation 40, CD154: CD40 ligand, CD62P: P‐selectin, PSGL1: P‐selectin glycoprotein ligand 1). Created with BioRender.com. See also.^[^ ^126^ ^]^

Platelets are important mediators of immune response to biomaterials. They can recognize and bind to biomaterials through various receptors, such as toll‐like, glycoprotein, or complement receptors, and become activated.^[^ ^126^ ^]^ Activated platelets can modulate MP recruitment, activation, and polarization by direct cell‐cell contact, secreted cytokines, chemokines,^[^ ^127^, ^128^ ^]^ and EVs (**Figure** **6**).^[^ ^129^, ^130^, ^131^ ^]^ MP polarization toward M2 phenotype can be influenced by platelet‐derived biomolecules such as platelet‐derived growth factor (PDGF) and transforming growth factor beta (TGF‐β).^[^ ^128^, ^132^ ^]^

##### Neutrophil Response

Neutrophils infiltrate the biomaterial‐blood interface following implantation. Their main function is to eliminate foreign bodies, necrotic tissues, and bacteria by phagocytosis.^[^ ^133^ ^]^ However, when they encounter a foreign body larger than 350 nm, they undergo activation by binding of β2 integrins of the neutrophils to arginylglycylaspartic acid (RGD) and Pro‐His‐Ser‐Arg‐Asn (PHSRN) motifs on adsorbed fibrinogen or by recognition of damage‐associated molecular patterns (DAMPs) on apoptotic or necrotic cells.^[^ ^134^ ^]^ This results in the activation of nuclear factor kappa‐light‐chain‐enhancer of activated B cells (NFκB) and mitogen‐activated protein kinase (MAPK) signaling pathways, which induce the expression of pro‐inflammatory mediators.^[^ ^135^, ^136^ ^]^ These mediators attract monocytes to the site of implantation, and polarize them into M1 phenotype, which amplify the inflammatory response.^[^ ^137^ ^]^

In normal wound healing, neutrophils typically undergo apoptosis within one to two days and release molecules that inhibit further neutrophil recruitment and induce MP polarization to M2 phenotype, which facilitate tissue repair and biomaterial integration.^[^ ^138^, ^139^ ^]^ However, when the presence to foreign body continues, such as in case of irritating implant, the lifespan of neutrophils is prolonged up to four days to sustain chronic inflammation, and the regenerative process is compromised.^[^ ^138^ ^]^ In response to foreign surfaces, neutrophils release NETs, which are composed of DNA, histones, proteases, complement components, and other proteins and ions.^[^ ^99^, ^101^ ^]^ NETs contribute to biomaterial‐induced thromboinflammation.^[^ ^101^ ^]^ The histones in NETs interfere with natural anticoagulant mechanisms, and the DNA in NETs provides a surface for coagulation contact pathway activation, leading to increased thrombin generation and complement activation.^[^ ^101^ ^]^

##### Monocyte and Macrophage Response

Monocytes are recruited and activated by the complement system^[^ ^140^ ^]^ and chemokines are released by activated neutrophils, such as macrophage inflammatory protein‐1 (MIP‐1),^[^ ^141^ ^]^ and interferon gamma (IFN‐γ).^[^ ^142^ ^]^ They can adhere directly to biomaterial surface, become activated, and secrete pro‐inflammatory cytokines (**Table** **1**). They also undergo the upregulation of their procoagulant tissue factor, which can enhance coagulation and inflammation,^[^ ^143^ ^]^ and may lead to implant degradation and failure.^[^ ^101^ ^]^ MPs can adopt M1 (pro‐inflammatory) or M2 (anti‐inflammatory or pro‐regenerative) phenotype.^[^ ^144^, ^145^ ^]^ M1 cells produce reactive oxygen (O_2_) species (ROS), nitric oxide (NO), and pro‐inflammatory cytokines, such as tumor necrosis factor alpha (TNF‐α), IL‐6 and IL‐12,^[^ ^144^, ^145^ ^]^ which can lead to tissue damage.^[^ ^146^, ^147^ ^]^ M2 cells produce anti‐inflammatory cytokines, such as IL‐10 or TGF‐β. MP polarization toward M2 phenotype can be induced by IL‐4, IL‐10, and IL‐13,^[^ ^147^ ^]^ which also decrease the secretion of pro‐inflammatory cytokine by monocytes.^[^ ^148^, ^149^ ^]^ In addition to regeneration, M2 cells also promote angiogenesis, and immunosuppression.^[^ ^150^ ^]^ During an inflammatory phase, both M1 and M2 cells exist concomitently, and the balance between M1 and M2, known as the M1/M2 ratio, plays a crucial role in wound healing and tissue regeneration. This ratio is influenced by the chemical and physical characteristics of biomaterials.^[^ ^151^ ^]^ The balance between M1 and M2 MPs is crucial for determining the outcome of response to biomaterials.

**Table 1:** Macrophage (MP) type 1 and type 2 stimulation, receptors, secreted factors, and function.^[^ ^144^, ^152^ ^]^

	M1 MP	M2 MP
Stimulation	IL‐1, IL‐6, IL‐12, IFN‐𝛾, TNF‐𝛼, LPS, HMGB1	IL‐4, IL‐6, IL‐10, IL‐13, GC, AMP, LPS, TNF‐𝛼, TLR, TGF‐𝛽
Receptors	Surface: MHC‐II, CD86, CD80, CD68, CD36, IL‐1R, TLR‐2, TLR‐4, iNOS Intracellular: IRF3, IRF5, STAT1, STAT5, HIF‐1α	Surface: CD206, CD163, CD209, CD86, CXCR1, CXCR2, Dectin‐1, IL‐10R, IL‐6R, TLR‐1, TLR‐8 Intracellular: Arginase I, STAT3, STAT6, IRF4, KLF4, JMJD3, PPARδ, PPAR‐γ, cMaf, cMyc
Secretion	Cytokines: TNF‐α, IL‐1α, IL‐1β, IL‐6, IL‐12, IL‐23 Chemokines: CXCL9, CXCL10, CXCL11, CXCL16, CCL5	Cytokines: TGF‐β, IL‐10, IL‐6 Chemokines: CCL1, CCL16, CCL17, CCL18, CCL22, VVL24, CXCL13 Growth factor: CSF‐1, VEGF, EGF, PDGF
Function	Pro‐inflammatory Arteriosclerosis Tissue damage Infection protection Anti‐cancer immunity Autoimmune diseases	Anti‐inflammatory Tissue regeneration and repair Angiogenesis and immunomodulation Pathogen recognition and killing Phagocytosis Tumor formation and progression

##### Fibroblast and Myofibroblast Response

**Figure 7:**
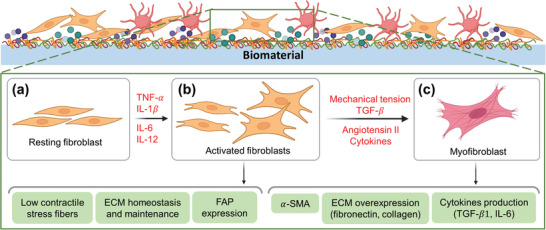
Schematic showing: a) Quiescent (resting) fibroblasts, which can be subjected to pro‐inflammatory cytokines such as tumor necrosis factor alpha (TNF‐α), interleukin 1 beta (IL‐1β), IL‐6), and IL‐12, and differentiate to activated fibroblasts b), leading to the expression of fibroblast activation protein (FAP), which can cleave collagen, gelatin, fibronectin, and cytokines, leading to modulation of their biological functions. Activated fibroblasts differentiate to myofibroblasts c) under the influence of neighboring activated fibroblasts via the effect of TGF‐β, Angiotensin II, and cytokines such as IL‐1α/β, and mechanical tension. Myofibroblasts have extensive stress fiber network (α‐SMA). Myofibroblasts can express extracellular matrix (ECM) proteins such as fibronectin and collagen and cytokines such as transforming growth factor beta (TGF‐β) and IL‐6, which will reinforce myofibroblast reaction to foreign body, leading to fibrosis. α‐SMA: alpha‐Smooth Muscle Actin. Created with BioRender.com.

Fibroblast reaction may end in fibrosis (**Figure** **7**), which is usually linked with hypoxia and hypoxia‐inducible factor 1a (HIF‐1a). Monocytes and MPs release TGF‐β,^[^ ^153^ ^]^ which promotes fibroblast activation, proliferation, and differentiation into myofibroblasts.^[^ ^153^, ^154^ ^]^ MPs also orchestrate the local inflammatory responses that sustain fibrotic responses and prevent the emergence of pro‐resolution pathways.^[^ ^153^ ^]^ Activated fibroblasts have highly expressed fibroblast activation protein (FAP), which can cleave collagen, fibronectin, and cytokines, leading to modulation of their biological functions.^[^ ^155^ ^]^ At later stage, myofibroblasts may contribute to fibrosis by producing excessive amounts of extracellular matrix (ECM) proteins, such as collagen and fibronectin, that disrupts the normal tissue architecture and impair organ function.^[^ ^156^, ^157^, ^158^ ^]^ Myofibroblasts may also persist and prevent the resolution of fibrosis.^[^ ^159^ ^]^

### 2.2 Specific Immunity

The specific adaptive immune response is the reaction of the immune system to specific antigens that are recognized by specialized receptors on B and T cells.^[^ ^160^ ^]^ B cells produce antibodies that bind to antigens and neutralize them, and T cells can directly kill abnormal cells or help other immune cells to perform their functions.^[^ ^161^ ^]^ Specific immune reactions are rarely seen to occur with commonly used biomaterials.^[^ ^162^ ^]^ However, there are rare reports in literature on allergic reactions to metals, such as nickel, cobalt, and chromium, that are used in orthopedic and dental implants.^[^ ^163^, ^164^, ^165^ ^]^ These reactions can cause inflammation and implant failure.^[^ ^164^, ^165^ ^]^ For newly developed biomaterials, testing for allergenicity must be conducted as a part of biomaterial characterization before moving forward with the clinical application of the biomaterial.^[^ ^166^, ^167^ ^]^ These tests include checking for T cell activation and cytokine production to judge the absence or presence of specific immunity.^[^ ^168^ ^]^

#### 2.2.1 Molecular Events

Biomaterials can stimulate or suppress the activation and differentiation of B and T cells by affecting their signaling pathways,^[^ ^169^ ^]^ transcription factors,^[^ ^170^ ^]^ or cytokine production.^[^ ^169^ ^]^ If the adaptive immune response against a biomaterial does not impair wound healing despite the production of antibodies, the material is considered “immunologically compatible”.^[^ ^171^ ^]^

#### 2.2.2 Cellular Responses

In addition to modulating the innate immune system, T cells also regulate adaptive immunity.^[^ ^172^ ^]^ T cell‐based specific immunity plays a role in modulating MP response to biomaterials.^[^ ^173^ ^]^ Ideally, T cell response to a biomaterial should be diverted away from FBR towards remodeling and regeneration.^[^ ^174^ ^]^ Specific immune reaction involves the APCs which are the large class of DCs that are found in most body tissues. DCs can also affect the expression or function of major histocompatibility complex (MHC) molecules, co‐stimulatory molecules, or T cell receptors (TCRs) that are involved in antigen recognition.^[^ ^175^ ^]^ They recognize antigens and present them to T cells via MHC molecules, leading to T cell activation and proliferation. This results in the production of specific T cells and B cells that can target the antigen and eliminate it. Biomaterials can also influence the polarization of T cells into different subsets, such as Th1, Th2, Th17, or regulatory T cell (Treg), that have different effector functions.^[^ ^176^ ^]^ T cells are therefore, a part of specific reaction that may occur in the presence of implants.^[^ ^177^ ^]^

### 2.3 Bridging of Nonspecific and Specific Immunity

DCs are the most efficient APCs that activate naive T cells and link innate and adaptive immunity.^[^ ^178^ ^]^ The interaction of DCs with biomaterials seems to be crucial for the function of biomaterials and has become an important area of research.^[^ ^179^ ^]^ Specifically, biomaterials can influence DC function by altering their microenvironment, including factors like surface chemistry, stiffness, and topography. This change can modulate DC maturation, cytokine secretion, and antigen presentation, thereby affecting the immune response. This ability to influence DC behavior has significant implications for optimizing biomaterials in medical applications, including tissue engineering and immunotherapy.^[^ ^180^ ^]^ Lymphocytes are also involved in the progression of tissue response to chronic inflammation,^[^ ^181^ ^]^ that involves plasma and giant cells.^[^ ^182^ ^]^

## 3 Modulation of Immune Response to Biomaterials

Progress in this area moved from passive to active and intelligent management of tissue responses. The objective of immunomodulation has advanced from initially aiming to avoid tissue reactions by using bioinert materials, to preventing unwanted inflammatory reactions through surface modifications and anti‐inflammatory agents. Subsequently, the focus moved towards creating bioactive materials that bond with tissues for better integration and leveraging tissue reactions for beneficial outcomes such as guided tissue formation. The current emphasis is on modulating tissue responses towards healing and regeneration by engineering biomaterials with immunomodulatory properties, using advanced techniques to elicit selective immune responses. Details of these steps in evolution are discussed in the following subsections.

### 3.1 Evolution of Objectives of Immunomodulation

**Figure 8:**
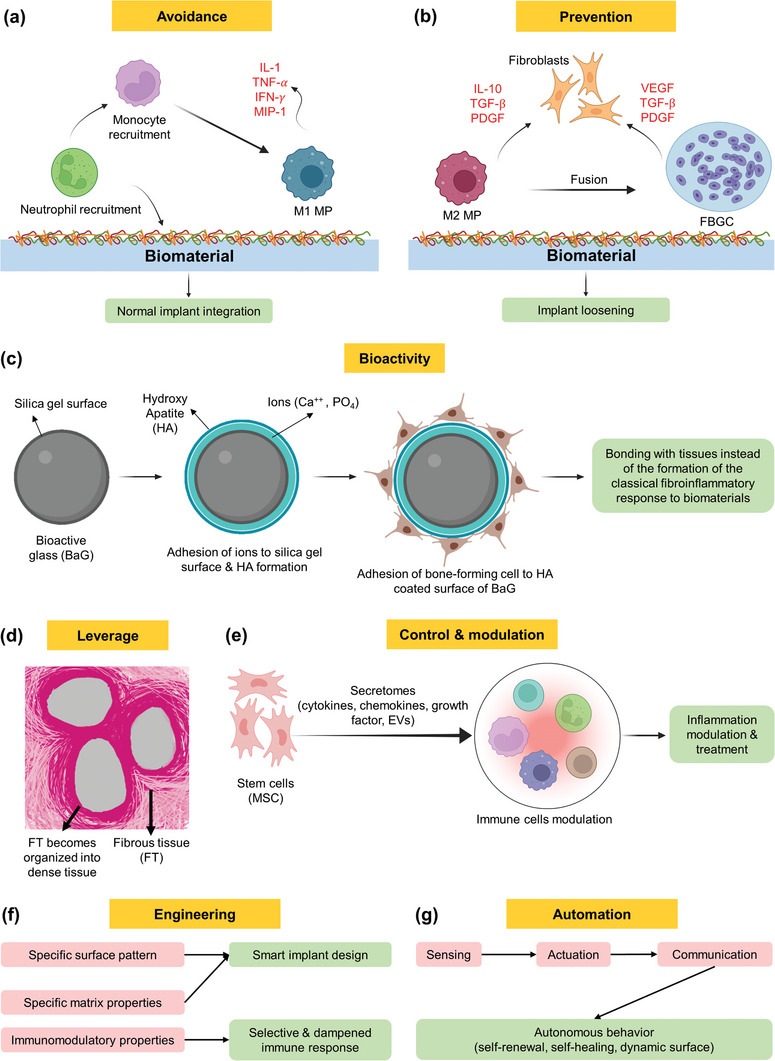
Schematic illustration showing the evolution of the objectives of desired immune responses and concepts: a) minimal tissue response to an inert biomaterial; b) corrosion products which lead to chronic inflammation, fibrous tissue formation, and implant loosening. c) the concept of bioactivity of materials such as bioactive glass (BaG) and the formation of an apatite layer that bons tissue to the implanted BaG; d) the concept of exploiting tissue responses into useful application such as fibrous tissue joint formation; e) control and modulation of tissue reactions; f) engineering of tissue response; g) and development of automated implants. (TGF‐β: transforming growth factor beta, TNF‐α: tumor necrosis factor alpha, IL: interleukin, IFN‐γ: interferon‐gamma, VEGF: vascular endothelial growth factor, PDGF: platelet‐derived growth factor). Created with BioRender.com.

The objective of research and development related to tissue responses varied through years and evolved with the increasing knowledge that has been gained and technologies that became available for us to embrace more intelligent and active role in making these interactions more controllable, useful and manageable using smart autonomous implants and therapy circuits in the future. In the following sections, we discuss how these stages in the management of tissue responses have evolved over time (**Figure** **8**).

#### 3.1.1 Avoidance of Tissue Reactions

One of the early objectives in the development of biomaterials was focused on avoiding tissue response. This led to the development of so‐called *bioinert* biomaterials (**Figure** 8a). Examples of bioinert biomaterials include metals (such as stainless steel, Ti, and cobalt‐chrome alloys), ceramics (such as alumina and zirconia), and polymers (such as ultra‐high molecular weight polyethylene).^[^ ^183^ ^]^ However, bioinert materials lack integration with the surrounding host tissue, which can result in implant instability and loosening. In fact, many of these materials were found to release ions or particles which can be toxic^[^ ^184^ ^]^ and; therefore, they cannot be considered absolutely bioinert in the human body.

#### 3.1.2 Prevention of Tissue Reactions

The next step in addressing tissue responses to materials followed the objective of preventing unwanted inflammatory tissue responses to biomaterials.^[^ ^14^ ^]^ Explored strategies included implant surface patterning,^[^ ^185^ ^]^ coating and functionalization,^[^ ^14^ ^]^ and the use of anti‐inflammatory agents,^[^ ^14^, ^186^ ^]^ or matching of implant mechanical properties to those of target tissue (**Figure** 8b).^[^ ^187^ ^]^ Most of these approaches however, have been explored on experimental level and were not translated to the clinic yet. Details of these approaches are discussed below in Section 3.3.

#### 3.1.3 Bioactivity, Favored Biomaterial‐Tissue Interface

The following step in approaching tissue responses focused on developing bioactive materials that can bond to surrounding tissues due to their bioactivity. A good example to demonstrate this is the BaG which forms an apatite layer that bonds it to tissues (**Figure** 8c). Another example is the reaction leading to the formation of oxide on the surface of Ti implants, which leads to improved osseointegration.^[^ ^188^ ^]^

#### 3.1.4 Useful Exploitation of Tissue Reactions

The next stage in research was guided by the idea of leveraging immune response to biomaterials, e.g., in inducing desired response, such as guided fibrous tissue formation,^[^ ^189^, ^190^ ^]^ and fibrous tissue joints (**Figure** 8d),^[^ ^191^ ^]^ studies of which reached the stage of clinical trial.^[^ ^192^ ^]^

#### 3.1.5 Modulation of Tissue Reactions

With more understanding of tissue responses, and new technologies that become available, the objective was focused on modulating tissue responses to biomaterials toward healing and regeneration (**Figure** 8e).^[^ ^123^, ^193^, ^194^ ^]^

#### 3.1.6 Engineering

Using advanced techniques, the objective of research moved toward engineering immune responses. Although challenging, it provides an opportunity for improving the function of implants (**Figure** 8f). One way to engineer the immune response to biomaterials is to empower them with immunomodulatory properties that can elicit selective immune responses depending on the application or situation. This can be achieved via various strategies including molecule release upon demand or in response to triggers.^[^ ^10^ ^]^ Other strategies include the use of certain surface patterns or matrix properties that lead to the activation, polarization, or differentiation of relevant cells.^[^ ^53^, ^195^ ^]^ Details of these approaches are discussed below in Section 3.3. Using this approach, it will be possible to enhance the durability and success of implants. Furthermore, this will enable the development of implants that can mimic native tissues in dynamicity, sensing and responding to changing circumstances.

#### 3.1.7 Automation

The ultimate aim of biomaterial development is to have an implant that can function like native tissues do, i.e., become self‐aware and autonomous (**Figure** 8g). To this end, implants need to integrate sensors for detecting aberrations from the norm in their surrounding environment.^[^ ^196^ ^]^ In addition, they need to integrate actuation and self‐repair capabilities. Few proof‐of‐principle reports were published so far.^[^ ^197^, ^198^ ^]^ This will also allow for possible fine tuning of installed implants.^[^ ^199^, ^200^ ^]^ Data relayed from sensors can be handled using artificial intelligence (AI) to assist implants to become more autonomous in dealing with tissue responses toward improved and sustained function.

Sensing should include general variables related to the state of healing such as inflammation and angiogenesis, which are associated with changes in pH and local O_2_ concentration.^[^ ^201^ ^]^ In addition, specific markers related to biomaterial, tissue type or function, or stage of healing^[^ ^202^ ^]^ need to be detected, either on the gene expression level or as secreted molecules or effect. Actuation can be chemical, physical, or biological, involving controlled drug release,^[^ ^203^ ^]^ electrical,^[^ ^204^, ^205^, ^206^ ^]^ magnetic,^[^ ^207^, ^208^, ^209^ ^]^ optical,^[^ ^210^, ^211^, ^212^ ^]^ or acoustic^[^ ^213^, ^214^, ^215^ ^]^ waves and systems, cellular elements such as stem cells,^[^ ^216^ ^]^ immune cells^[^ ^217^ ^]^ and other types of cells,^[^ ^218^ ^]^ or combinations of those approaches^[^ ^219^ ^]^ to achieve control over immune reactions to biomaterials. Stimuli‐responsive biomaterials can also be used for actuation, e.g., they can deliver magnetic nanoparticles that can carry drugs or bioactive molecules for modulating the immune response.^[^ ^220^, ^221^ ^]^

Autonomous implants of the future are envisioned to be independent of external control and they mimic more closely native tissues in the sense they can be aware of surrounding immune reactions,^[^ ^196^ ^]^ have the ability to modulate it and deal with issues independently. This will be achieved through proper integration of sensing, and actuating^[^ ^196^ ^]^ functions, and the use of smart materials such as self‐healing^[^ ^222^ ^]^ and bioresponsive materials^[^ ^223^, ^224^ ^]^ and dynamic surfaces^[^ ^224^ ^]^ into the implants.

### 3.2 Targets of Immunomodulation

Initiation, activation, and resolving of innate inflammatory reactions mediate a set of complex interactions between the various immune and non‐immune molecular and cellular components.

#### 3.2.1 Molecular Systems

**Figure 9:**
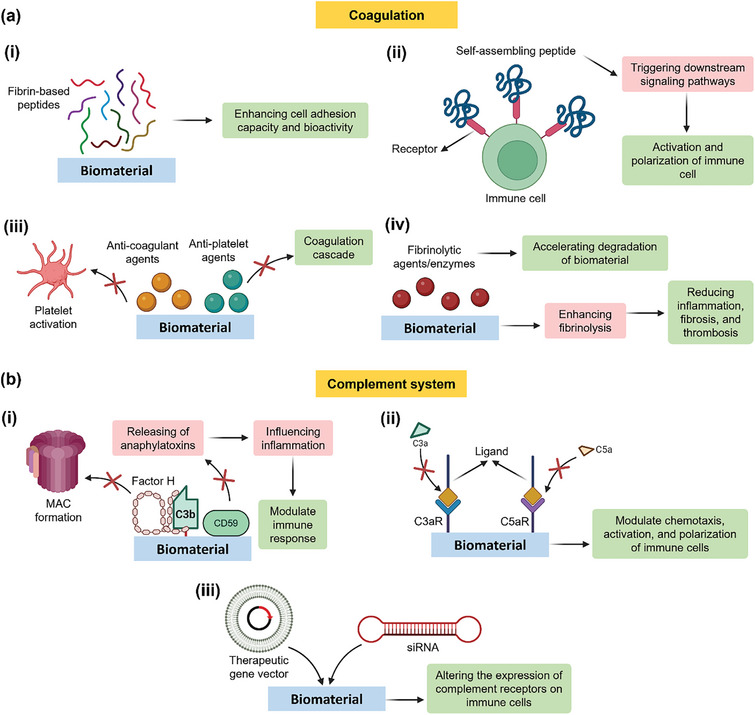
Schematic showing the molecular targets of immunomodulation and engineering. Targeting coagulation and fibrinolytic systems to modulate the immune response to implanted biomaterials a) by: i) using fibrin‐based peptides which lead to better cell adhesion and bioactivity of biomaterial; ii) leveraging self‐assembling peptides incorporate with immune cell's receptor, which trigger downstream signaling pathways and activation of immune cells; iii) incorporating anti‐platelet and anti‐coagulant agents resulting the prevention of platelet activation and inhibition of coagulant cascade; iv) subjecting the biomaterial by fibrinolytic agents/enzymes in order to fast the degradation process of biomaterial. Fibrinolytic agents enhance fibrinolysis which will result in reduced inflammation, fibrosis, and thrombosis. Targeting the complement system (b), by using complement inhibitors, factor H, and cluster of differentiation 59 (CD59), which will inhibit the membrane attack complex (MAC) formation and anaphylatoxins release (i). This will influence the inflammation and help to modulate the immune repones. ii) incorporating ligands for complement receptors compliment component 3 smaller element receptor (C3aR) and C5aR in order to modulate the chemotaxis and polarization of immune cells. iii) delivering gene therapy vectors and short interfering ribonucleic acids (siRNAs) to biomaterial in order to alter the expression of complement receptor on immune cells. Created with BioRender.com.

Fibrin has an array of binding sites that facilitate interactions with cell adhesion molecules and integrins important for coagulation, inflammation, and tissue repair. Therefore, modulation of immune response to biomaterials can be achieved by targeting fibrin binding or mimicking fibrin structure. Mimicking the structure and function of fibrin, by self‐assembling nanofibers, can be employed to improve biomaterial biocompatibility and bioactivity.^[^ ^225^, ^226^ ^]^ It is also possible to influence immune cell recruitment, activation, and polarization by targeting fibrin.^[^ ^74^ ^]^ Other strategies include the inhibition of the coagulation cascade and preventing the activation of platelets.^[^ ^227^ ^]^ The fibrinolytic system also offers targets for intervention to modulate tissue response (**Figure** **9**a). Almost all immune cells have at least one of plasminogen receptors that allows plasmin formation on the cell surface that in turn modulates immune cell behavior.^[^ ^228^ ^]^ Another target is enhancing fibrinolysis, which leads to reduced thrombosis, inflammation, and fibrosis.^[^ ^229^ ^]^

One target can be the inhibition of the complement cascade by using agents that can bind specific complement components or regulators. By reducing the complement activation and the subsequent inflammatory response, biomaterials integration with the host tissue can be enhanced. Another target related to the complement system is complement receptors, for which ligands integrated to biomaterials can be used for binding these receptors and modulate the immune response (chemotaxis, activation, and polarization of immune cells) to implants.^[^ ^230^, ^231^, ^232^ ^]^ Other biomaterials can alter the expression of complement receptors on immune cells,^[^ ^233^, ^234^ ^]^ which influence their recruitment, differentiation, and function, leading ultimately to modulated immune response (Figure 9b).^[^ ^233^, ^234^, ^235^ ^]^

#### 3.2.2 Cellular Systems

##### Targeting Platelets

Targeting platelet function, adherence, activation, or stabilization of their content to prevent the release of active molecules is a possible strategy to modulate the immune response to biomaterials. For example, some biomaterials enhance the function of platelets, leading to regeneration by providing growth factors.^[^ ^236^ ^]^ In contrast, others inhibit platelets and prevent thrombosis, leading to reduced inflammation caused by platelet‐derived mediators.^[^ ^237^, ^238^ ^]^ A specific example is biomaterials that modulate membrane integrity or intracellular signaling of platelets to control the release of active molecules, which directly impacts their role in balancing inflammation and tissue repair.^[^ ^239^, ^240^ ^]^ By influencing the platelet behavior, biomaterials can thus impact the balance between inflammation and tissue repair processes.^[^ ^240^ ^]^

##### Targeting Macrophages

Targeting MP polarization, metabolism and fate, is a potential strategy to modulate the immune response in various diseases. For instance, polylactide degradation products^[^ ^241^, ^242^, ^243^, ^244^ ^]^ and polyethylene wear particles^[^ ^245^, ^246^ ^]^ have been shown to upregulate glycolytic flux and mitochondrial respiration in surrounding immune cells, resulting in a pro‐inflammatory phenotype. Accordingly, methods aimed at modulating glycolytic reprogramming in the biomaterial microenvironment result in immunomodulatory outcomes.^[^ ^244^ ^]^ Furthermore, strategies to inhibit the pro‐inflammatory M1 phenotype have successfully reduced tissue damage and fibrosis in experimental models.^[^ ^247^ ^]^ Conversely, enhancing regeneration by promoting MP polarization toward proregenerative M2 phenotype has improved healing in diverse applications, including the treatment of wounds,^[^ ^248^ ^]^ myocardial infarction,^[^ ^46^, ^249^, ^250^ ^]^ spinal cord injury,^[^ ^146^ ^]^ and peripheral nerves.^[^ ^251^, ^252^ ^]^

**Figure 10:**
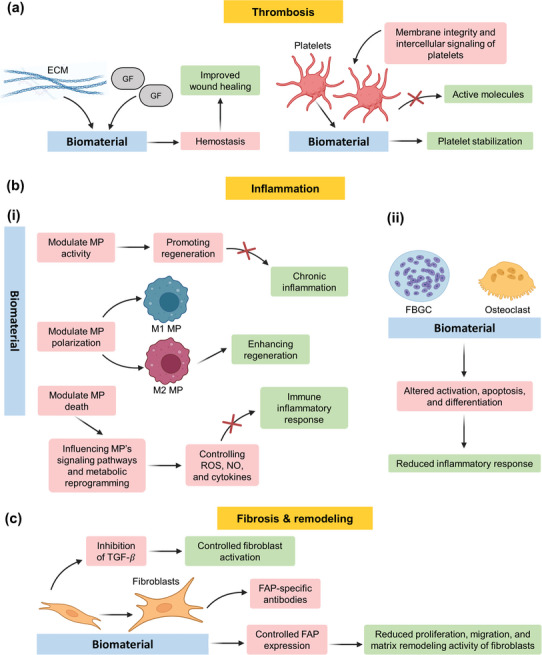
Schematic showing the cellular systems of targets of immunomodulation and engineering: One of the methods is targeting thrombosis, which includes providing growth factors (GFs) and extracellular matrix (ECM) to promote hemostasis, leading to improved wound healing and regeneration. Thrombosis targeting also includes modulating the stabilization of platelet and prevention of release of active molecules from platelets by affecting their membrane integrity or intracellular signaling (a). The targets also include interfering with the pathways of inflammation (b) by modulating macrophage (MP) activity, polarization, and death (i), giant cell and osteoclast formation and function (ii), and fibrosis (c). Created with BioRender.com.

By manipulating the MP activation and polarization, one can influence the balance between inflammation and regeneration.^[^ ^253^ ^]^ Furthermore, biomaterials designed to induce M1 polarization through toll‐like receptor (TLR) ligands have been shown to stimulate phagocytosis and enhance host defense in cancer therapy.^[^ ^254^ ^]^ Metabolic reprogramming of MPs is a potential target for immunomodulation of biomaterials and a key feature of MP polarization and function that can be influenced by biomaterials.^[^ ^242^, ^243^, ^244^, ^255^, ^256^, ^257^, ^258^, ^259^, ^260^ ^]^ For example, biomaterials that promote glycolysis in MPs can enhance their pro‐inflammatory phenotype and facilitate infection clearance or tumor eradication.^[^ ^255^, ^261^ ^]^ In contrast, biomaterials that promote fatty acid oxidation in MPs can enhance their anti‐inflammatory phenotype and facilitate tissue repair or regeneration.^[^ ^262^, ^263^, ^264^ ^]^ Biomaterials that modulate the survival or death of MPs can also affect their clearance or persistence in the tissue. Similarly, one can modulate the death of MPs by affecting their signaling pathways or metabolic reprogramming.^[^ ^265^, ^266^ ^]^ This can regulate the immune response by controlling the production of ROS, nitric oxide, and cytokines (**Figure** **10**b‐i).

##### Targeting Foreign Body Giant Cells

Foreign body giant cells (FBGCs) and osteoclasts represent possible targets for the modulation of immune reaction.^[^ ^182^ ^]^ Targets may include FBGC formation,^[^ ^8^, ^267^ ^]^ function,^[^ ^8^ ^]^ and fate,^[^ ^99^ ^]^ that can be influenced using chemical and physical^[^ ^268^, ^269^, ^270^ ^]^ strategies. This was found to reduce the inflammatory response and tissue damage caused by FBR (**Figure** 10b‐ii).^[^ ^99^ ^]^

##### Targeting Fibroblasts

Targeting fibroblast activation and function represents a potential objective to modulate the immune response to biomaterials. For instance, inhibition of TGF‐β, a key cytokine in fibrosis and scar formation, has been explored.^[^ ^271^ ^]^ Another strategy involves the control of FAP, which is highly expressed by activated fibroblasts and cancer‐associated fibroblasts.^[^ ^272^, ^273^ ^]^ There is a study, which demonstrated that targeting FAP can enhance bone regeneration.^[^ ^274^ ^]^ However, more research is needed to understand the exact mechanisms and potential side effects of such intervention (**Figure** 10c).

##### Targeting Specific Immunity

Targeting specific immunity cells is important in engineering immune response to cancer. Immune engineering can address different aspects of the specific adaptive immune response, such as antigen presentation and recognition, activation and differentiation of B and T cells, and memory and recall of B and T cells.^[^ ^160^, ^275^ ^]^

### 3.3 Strategies for Achieving Immunomodulation and Engineering

To modulate reactions to biomaterials,^[^ ^113^ ^]^ different physical, chemical, and biological strategies have been investigated (**Table** **2**). These methods can be used separately or in combination to influence biomaterial interactions with cells, proteins, and other molecules.

**Table 2:** Summary of strategies developed to address tissue reactions to biomaterials, their types, modes of delivery, targets, advantages and disadvantages, with explanation of types of studies used, and sources.

Type	Agent	Mechanism of action	Study type	Outcome	Advantages	Disadvantages	Refs.
A. Physical strategies							
Shape	Spherical, rod‐shaped, and star‐shaped nanoparticles	Shape‐dependent differential uptake and activation of MPs	In vivo, in vitro	Spherical nanoparticles induced a mild inflammatory response, rod‐shaped induced a strong pro‐inflammatory response, and star‐shaped induced a strong anti‐inflammatory response	Specific shapes can be designed to modulate the immune response	Shape‐specific responses may vary and need precise control	[276, 277, 278]
Surface	Roughness, porosity, wettability	Modulation of platelet adherence/activation and cell behavior through surface modifications	In vitro, in vivo, clinical trials	Can either reduce or enhance platelet adhesion and activation. Reduction prevents the formation of a thick fibrin layer around the implant and better tissue integration	Surface properties can be engineered to achieve desired interactions with immune cells	Requires in‐depth understanding and control of surface characteristics	[51, 279]
	Patterning/ topography	Nano‐/micro‐ patterns, pillars can direct the alignment and orientation of fibroblasts, polarization MPs to anti‐inflammatory phenotype, and enhance DC activation. They also can promote osteogenic differentiation of mesenchymal stem cells (MSCs).	In vitro, in vivo	Improved implant integration, prevent biofouling, infection, and thrombosis	Enhance cell adhesion and proliferation	Limited predictability	[53, 309, 310, 312]
	Functionalization	– Using specific chemicals agents or molecules such as peptides or cytokines that can bind to receptors or ligand on immune cells and tissue cells. – Dual functionalization of electrospun resorbable elastomers with heparin for an anti‐thrombogenic role and IL‐4 for an anti‐inflammatory. Enhances cell adhesion and migration, and M2 polarization	In vitro, in vivo	Enhanced tissue regeneration	Controlled immune response, enhanced biocompatibility	Long‐term stability, high cost and complexity of the manufacturing	[315, 317]
	Potential	Adjust surface potential, which can aid in regulating the expression of adhesion molecules. Coupling biomaterial with light, electric, magnetic, or ultrasound sources, or coating with polydopamine (PDA), or with COO‐ functionalized NPs to modify the surface potential, inhibiting PI3K‐Akt‐mTOR signaling pathway, activating FAK pathway and expressing adhesion‐related genes	In vitro, in vivo, clinical trials	Activating M2 polarization, regulating the expression of adhesion molecule	Reduced chance of immune response	Complexity of surface engineering, long‐term stability, unpredictability	[84, 318, 319, 320, 321]
	Charge	– Negatively charged surfaces attract positively charged proteins such as fibronectin and vitronectin – Positively charged surfaces enhance cell attachment and proliferation, may induce activate complement and coagulation system	In vivo, in vitro	Mediating cell adhesion and spreading, enhancing the cell proliferation, inducing inflammatory response and thrombosis	Controlled immune response, improved cell adhesion and proliferation	It should be carefully controlled to achieve the desired biological response	[52, 322]
Structure	Porosity, pore size, and poreinterconnectivity	Influences cellular infiltration, differentiation, and immune responses	In vitro, in vivo, clinical trials	Can enhance tissue ingrowth and vascularization and reduce inflammatory response	Structural parameters can be optimized for specific applications	Trade‐offs between mechanical properties and biological integration	[282, 283, 284, 285, 286, 287, 288, 419, 420]
Electrical, magnetic and magnetoelectrical properties	Biomaterials with inherent electrical properties	Modulation of immune cell activation, polarization, cytokine production, and antigen presentation through the alteration of surface charge properties	In vitro, in vivo	– Negatively charged polymers reduced adsorption of pro‐inflammatory proteins and decreased neutrophils and MPs adhesion and activation. – Positively charged PLGA nanoparticles enhanced DCs uptake and maturation by activating the NF‐κB pathway.	Ability to modulate immune response by altering biomaterial surface charge, potential for targeted immunomodulation	The complexity of immune responses to different charges, potential unintended effects on other cellular or tissue‐level processes	[294, 298, 299, 300, 301]
	Electrical stimulation	Modulates MP phenotype towards M2 and reduces M1 phenotype, promoting wound healing	In vivo, in vitro	Enhanced wound healing due to increased M2 phenotype and reduced M1 phenotype in wounds	Non‐invasive, can be targeted to specific wound sites, promotes tissue regeneration	Equipment and expertise required for application, effects may vary depending on the parameters used (intensity, duration, frequency)	[297]
	Optogenetic stimulation	Emission of light of varying intensity and frequency to stimulate or inhibit cells expressing light‐sensitive proteins, modulating immune responses	In vitro, in vivo	Modulation of immune responses through the activation or inhibition of targeted cells	Precise control over stimulation parameters, ability to target specific cell types, non‐invasive	Requires genetic modification of cells to express light‐sensitive proteins, specialized equipment, and expertise	[291, 292]
	External magnetic stimulation	Application of a magnetic field induces mechanical forces on immune cells (e.g., DCs) containing magnetic NPs, leading to altered cellular functions such as increased expression of co‐stimulatory molecules and cytokines, and enhanced ability to stimulate T cell proliferation and activation	In vitro, in vivo	Enhanced expression of co‐stimulatory molecules and cytokines by DCs Increased ability of DCs to stimulate T cell proliferation and activation	Targeted modulation of immune cell function, potential for drug delivery and immune response modulation	Requires careful control of magnetic field application, potential concerns with biocompatibility and long‐term effects of magnetic NPs	[220, 302, 304]
	External stimulation of magnetoelectric nanocomposites	Application of magnetic fields to magnetoelectric materials generates electric fields that can influence the electrophysiology and biochemistry of immune cells, as well as other cell types like bone marrow stromal cells and endothelial cells	In vivo, in situ	Increased expression of osteogenic and angiogenic proteins by bone marrow stromal cells and endothelial cells Improved bone formation and vascularization in bone defects	Ability to modulate cell function and promote tissue regeneration non‐invasively through magnetic field application	Complexity in designing and applying magnetoelectric materials, potential safety issues and adverse effects need careful evaluation	[305, 306, 307, 308]
Mechanical Properties	Matching the mechanical properties of the biomaterial with those of the host tissue	– Reduces fibrotic reactions by matching the mechanical softness of brain tissue – Can lead to pro‐fibrotic reactions due to mismatch with the mechanical properties of surrounding tissue	In vivo, in vitro	– Reduced fibrotic reaction in brain implants, – Increased risk of fibrosis around the implant	Better integration with soft tissues, reduced risk of fibrosis, may provide necessary support and stability for certain applications	May not be suitable for load‐bearing applications, risk of fibrosis, stress shielding, and weakening of adjacent bone tissue	[69, 332, 333, 334, 335, 336]
		The degradation process changes the mechanical properties over time, potentially matching the healing tissue's mechanical properties	In vitro, in vivo	Potential for improved tissue healing due to gradual transfer of load and changing mechanical properties	Biodegradability, potential for mechanical property matching over time	Degradation can be associated with chronic inflammation, precise control of degradation rate is challenging	[422]
	Immunoengineering by specific mechano‐stimulation	Influences T and B lymphocyte functions including DNA regulation, morphology changes, migration, substrate discrimination, target cell killing, and antigen uptake through the application of mechanical forces	In vitro, in vivo	Modulation of lymphocyte behavior and immune response, potentially enhancing the effectiveness of immune responses	Non‐invasive modulation of immune responses, potential for precise control of lymphocyte activity	Requires detailed understanding of force parameters for desired outcomes, potential variability in responses between individuals or tissues	[325]
	Mechano‐responsive materials	These materials respond to mechanical stress or stimuli by altering their properties, such as healing themselves, changing color, stiffening under strain, thinning under shear, or directly delivering mechanical stimuli to cells	In vitro, in vivo	Self‐healing and mechano‐responsive biomaterials enhance repair, diagnostics, and tissue mimicry, supporting regeneration and functionality	The dynamic functionality of these materials allows for innovative applications in biomedical engineering, including tissue engineering, regenerative medicine, and biosensing	complexity of designing materials that can reliably respond to mechanical stimuli in a predictable manner, potential biocompatibility issues	[325, 326, 327, 328, 330, 331]
B. Chemical strategies							
Pharmaceutical							
Steroids	Dexamethasone (DEX)	Blocks inflammatory mediators, decreases inflammatory cell releases, suppresses fibroblast proliferation	In vitro, in vivo, clinical trials	Suppressed arterial restenosis, mitigated astrocytic response, inhibited pro‐inflammatory cytokines	High potency and specific action	Adverse effects like osteoporosis and hyperglycemia when used systemically	[341, 342, 344, 403]
Non‐steroidal anti‐inflammatory drugs (NSAIDs)	Celecoxib	Modulates inflammation, inhibits cyclooxygenase‐2 (COX‐2) leading to reduced production of PGs	In vivo, clinical trials	Effects on osseointegration, enhanced bone formation with selective EP4 agonist	Broad availability, diverse options	Potential impairment of bone healing at non‐selective dosages	[346, 347, 348, 349, 406, 407]
	Ibuprofen	Inhibits COX‐1 and COX‐2, PG and thromboxane synthesis, decreases inflammation	In vivo, clinical trials	Improved joint mobility, alleviated fever	Less cardiovascular (CV) risk and gastrointestinal (GI) toxicity, rapid onset	Short half‐life	[345, 405, 423]
	Diclofenac sodium	Inhibits both COX‐1 and COX‐2 but more effective on COX‐2 leading to reduced PGs	In vivo, clinical trials	Alleviated fever	Versatility, rapid onset of action	GI & CV side effects, renal toxicity	[34, 350, 404, 424]
Other anti‐inflammatory drugs	Doxycycline	Inhibits matrix metalloproteinases (MMPs), protease‐activated receptor‐2 (PAR2) activation, leukocyte chemotaxis, nitric oxide synthases (NOS) levels, IgE pathway	In vivo, clinical trials	Enhanced implant integration in dental sockets and rat tibia, anti‐inflammatory effects through interleukin‐10 (IL‐10) regulation	Multiple mechanisms of action, additional benefits like antimicrobial properties	Varied efficacy, potential side effects depending on the drug and application method	[356, 357, 414]
	Beta blockers	Reduction of the TNF‐α and IL‐10 level	Clinical trials	Modified dysregulated cytokine network in DCM	Availability, effective for heart and circulatory condition	Possible side effects such as cold hands or feet and light‐headedness	[358]
Antiproliferative drugs	Sirolimus (Rapamycin)	Binds to FKBP‐12 leading to inactivation of mTOR	In vivo, clinical trials	Suppressed cytokine‐mediated T cell proliferation, halting progression of cell cycle from G1 to S phase	Improving renal function, useful in specific applications like stents, decrease autoimmunity	Increase in triglyceride	[360, 361, 413]
	Paclitaxel	Hyper‐stabilizing microtubules, reprograms tumor‐associated macrophages (TAMs)	In vitro, in vivo, clinical trials	Reduced restenosis, inhibits vascular smooth‐muscle‐cell proliferation	Broad spectrum, combination capability	Potential cytotoxicity at high doses, drug interaction	[362, 363, 412]
	Mitomycin C (MMC)	Inhibition of mitosis and protein synthesis affecting MPs, fibroblasts and lymphocytes	In vivo	Inhibited MP and fibroblast proliferation	Bio‐reductive alkylation	Careful monitoring needed for MMC due to side effects	[365]
	Azithromycin	Leads to M2 polarization	In vivo	Enhanced bone formation	Targeted immunomodulation, enhanced tissue integration	Specific to certain applications, long‐term effects and biocompatibility need further investigation	[367]
	MMP inhibitors (e.g., of MMP‐1, ‐8, ‐13, and ‐18)	Reduced formation of foreign body giant cells (FBGCs) by the fusion of MPs	In vitro	Reduction in the formation of FBGCs, which may decrease inflammatory responses to biomaterials	Direct targeting of MMPs involved in inflammatory processes	Potential off‐target effects, long‐term impact on tissue remodeling and healing needs careful consideration	[368]
Biomolecules							
Peptides	MP‐colony stimulating factor	Bind to cell surface receptors, modulate immune cell activation and polarization	In vitro, in vivo	Improved biocompatibility, increased M2 phenotype, decreased pro‐inflammatory cytokines, enhanced bone regeneration	High specificity, minimal side effects	Potential challenges with stability, delivery, and cost‐effectiveness	[44]
	Derivative of Itaconate	Modulation of inflammation and enhancement of bone regeneration	In vivo	Improved bone regeneration in cranial bone defects	Potential anti‐inflammatory effects, enhanced bone healing and regeneration	Specificity of action and delivery methods need to be optimized, potential variability in efficacy depending on the application site	[374]
	Fibrin‐peptides	Trigger the activation and polarization of immune cells	In vitro, in vivo	Potential modulation of the immune response, enhanced tissue integration and healing	Targeted action at the site of biomaterial implantation, potential for enhancing biomaterial integration	Challenges with peptide stability and delivery, the need for careful design to ensure specificity and efficacy	[226]
Cytokines	TGF‐β, IFN‐γ, TNF‐α	Facilitate cell activity, stimulate cell movement to injury sites, enhance cell differentiation, activation, and survival	In vitro, in vivo	M2 polarization by IL‐4, IL‐10 improves anti‐inflammatory pathways, lentiviral transmission of IL‐10 for MP polarization	Directly target specific immune pathways, potential for precise immunomodulation	Short half‐life, challenges with delivery and maintaining effective concentrations	[10, 382]
	IL‐4	M2 polarization, suppresses expression of inflammatory cytokines	In vitro, in vivo	Controlled release can tune the early inflammatory process	Useful for M2 polarization, directly targets specific immune pathways	Short half‐life limits its use	[377, 378]
	IL‐13	Effects on B cells and monocytes, inhibits cytokine inflammatory products	In vitro, in vivo	Inhibitory effects on inflammatory cytokines, modulates immune cell activity	Can modulate immune response, specifically affecting B cells and monocytes	Specifics of delivery and efficacy need to be well understood	[379]
	IL‐10	Suppression of inflammatory cytokines, lentiviral transmission for M2 polarization	In vitro, in vivo	Induces M2 phenotype polarization, improves anti‐inflammatory pathways	Potential for precise immunomodulation through lentiviral transmission	Challenges with viral vector use, maintaining effective concentrations	[380, 381]
	IL‐6	Improves anti‐inflammatory pathways in GC‐induced monocytes	In vitro, in vivo	Enhances anti‐inflammatory pathways	Can modulate immune response positively in the context of GC treatment	Context‐dependent effects, may not universally suppress inflammation	[380]
Glycosaminoglycans (GAGs)	Hyaluronic Acid (HA), chondroitin sulfate (CS), heparin (Hep)	Inhibit NF‐κB translocation, leading to suppressed production of pro‐inflammatory cytokines	In vitro	Anti‐inflammatory properties, modulation of immune cell activity	Naturally occurring, generally well‐tolerated	Efficacy may vary, possible degradation in vivo	[425]
Polynucleotides	DNA	Activation of immune checkpoints and the interaction with the DNA damage response (DDR) pathways	In vitro, in vivo, clinical trials	Regulation of immune responses, gene silencing	Broad potential applications in modulating immune responses	Delivery challenges, potential off‐target effects	[426, 427]
	RNA	Modulates the immune response primarily through RNA‐binding proteins (RBPs) that control gene expression and cell fate	In vitro, in vivo, clinical trials	Regulation of immune responses, potential for targeted gene expression	Rapid development potential, especially for vaccines and therapeutics	Stability issues, delivery challenges	[411, 426]
	siRNA	Targeted gene silencing, specific inhibition of protein expression	In vivo	Inhibition of TNF‐α expression in MPs, potential therapeutic applications	High specificity, potential for targeted therapeutic applications	Delivery challenges, potential immune stimulation	[429]
	Osteopontin (OPN) antisense oligodeoxynucleotide	Targeted gene silencing to accelerate wound healing and reduce scarring	In vivo	Intended to reduce OPN expression, limited success observed	Targeted action at gene expression level	Limited efficacy observed, challenges in delivery and achieving desired outcomes	[430]
	Type I collagen and TGF‐β1 antisense oligodeoxynucleotide	Silencing of target genes in hepatic stellate cells to reduce fibrogenesis	In vivo	Effective silencing of target genes, potential application in liver fibrosis	Targeted approach to fibrosis, potential for liver disease treatment	Delivery challenges, potential off‐target effects	[431]
Other biomolecules	Antibodies against PD‐L1	Enhance the antitumor immune response by tumor‐infiltrating T cells	In vivo	Enhanced antitumor immune response	Targeted action against tumor cells, potential for combination with other therapies	Need for careful design to avoid toxicity, long‐term biocompatibility concerns	[432]
	Ligands for complement receptors (Gold NPs with PD‐L1)	Enhance antitumor immune response by blocking PD‐1/PD‐L1 pathway, modulate immune cell chemotaxis and polarization	In vitro, in vivo	Modulation of immune responses, enhanced antitumor activity	Targeted action, potential for combination with other therapies	Need for careful design to avoid toxicity, long‐term biocompatibility concerns	[230, 232, 432]
Combinations	Cytokines (IL‐6, IL‐10) and GCs	Amplify anti‐inflammatory effect of GCs, investigate cytokine‐induced anti‐inflammatory monocyte subtypes	In vitro	Demonstrated that GC, but not cytokines, induced anti‐inflammatory monocyte subtype	Synergistic effects, enhanced specificity of action	Complex interplay may lead to unpredictable outcomes, requires thorough optimization	[380]
Other molecules							
Oxygen		Influence immune response (M1 and M2 cells) through HIF regulation	In vitro, in vivo	Modulation of immune cell phenotype, enhanced tissue oxygenation	Non‐pharmacological approach, broad applicability	Delivery and control of concentrations can be challenging, potential for systemic effects	[383, 385, 386]
Nitric oxide	Endothelially‐derived or biomaterial‐released NO	Exerts antiplatelet aggregation and anti‐inflammatory effects. Enhances the endothelial function	In vitro, in vivo	Prevention of platelet adhesion, aggregation, and recruitment to the growing thrombus, reduced inflammation at the site of implantation	It can enhance blood flow around implanted biomaterials, affecting T cell response	It can be either protective and toxic effects, inflammation potentiation	[387, 389]
Biomaterials							
Extracellular matrix (ECM)	Decellularized ECM	Facilitates constructive tissue remodeling without eliciting rejection	In vitro, in vivo	Shift in innate immune response from pro‐inflammatory to anti‐inflammatory, facilitated constructive and functional tissue remodeling	Natural composition close to the host tissue, promotes tissue integration	Potential variability in ECM composition and properties, risk of disease transmission if not properly processed	[390, 391, 392, 393, 394]
Bioceramics	Octacalcium phosphate (OCP)	OCP has a lower affinity to C3, influencing osteoclast formation, which is integral to bone resorption. This lower affinity to C3 may lead to a reduced stimulation of osteoclast formation, favoring bone regeneration processes and making OCP suitable for applications requiring reduced inflammatory responses and enhanced bone regeneration	In vivo	Better bone regeneration capacity compared to bioceramics with high affinity to C3 due to its low affinity to C3	Enhanced bone regeneration capabilities, potentially fewer inflammatory responses due to lower C3 affinity	Further research may be needed to fully understand and optimize its use in various clinical applications	[397]
	Hydroxyapatite (HAp)	HAp's immunomodulatory effects are mediated through its physical properties like size and shape, which affect cytokine production by immune cells such as DCs. The physicochemical characteristics of HAp, including surface functionalization, structural and textural characteristics (size, shape, surface topography), and incorporation of bioactive substances, can modulate the immune response, particularly by MPs, contributing to a favorable osteoimmune environment for bone regeneration	In vivo, in vitro	Potential reduction in bone regeneration capacity compared to bioceramics with low affinity to C3 due to its high affinity to C3	Widely used in bone repair and regeneration due to its compositional similarity to natural bone	High affinity to C3 may lead to increased osteoclast formation and potentially affect the bone regeneration process	[396, 398]
	Cerium‐containing ceramics	They switch their valence states between Ce+3 and Ce+4, leading to quenching free radicals, and promoting M2 MP polarization	In vitro	Reduction in inflammation and promotion of M2 MP polarization	Potential for enhancing the biocompatibility and anti‐inflammatory properties of ceramics used in scaffolds	Requires careful control of cerium content and distribution within ceramics to achieve desired effects	[399, 400]
	Monetite	Suppression of the expression of pro‐inflammatory cytokines (TNF‐α, IL‐1β, IL‐6) and chemokines (CCL2, CXCL10) by MPs, reduction in inflammatory cell infiltration (neutrophils, MPs), and increased recruitment of anti‐inflammatory cells (regulatory T cells, M2 MPs)	In vivo, in vitro	‐ Reduced infiltration of inflammatory cells increased recruitment of anti‐inflammatory cells, associated with enhanced bone regeneration and vascularization ‐ Associated with enhanced bone regeneration and vascularization	Potential anti‐inflammatory properties facilitating biomaterial integration with host tissue and promoting bone repair	Further research may be needed to fully understand the mechanisms and to optimize monetite and other calcium phosphate bioceramics for clinical applications	[394, 402]
	Molybdenum in bioactive glass (BaG)	Leads to MP polarization toward M2 phenotype by regulating mitochondrial function and immunometabolism in MPs	In vitro	enhancing tissue repair and regeneration	Modulates immunometabolism, promoting a regenerative environment	Specific effects and mechanisms require further elucidation, especially in humans	[409]
	Zinc oxide (ZnO) added to BaG	Leads to MP polarization toward M2 phenotype	In vitro, in vivo	Polarization of MPs towards M2 phenotype, contributing to bone repair	Enhances anti‐inflammatory responses, potentially improving scaffold integration and bone healing	Dosage and long‐term effects of ZnO incorporation need careful evaluation	[410]
Metals							
Controlling composition	Low‐nickel content in alloys	Reduces corrosion and minimizes inflammatory and allergic reactions triggered by metal ion release, enhancing biocompatibility compared to conventional stainless‐steel implants	In vitro, in vivo	Increased biocompatibility and reduced proneness to corrosion	Reduced risk of inflammation and allergic reactions, improved safety profile for patients sensitive to nickel	Potential challenges in mechanical properties and durability compared to traditional stainless steel	[434]
	Low‐carbon or nitrogen‐containing cobalt‐based alloys (e.g., Co‐Cr‐Mo, Co‐Ni‐Cr‐Mo)	Designed to reduce metallosis and mitigate the risk of inflammation and hypersensitivity reactions in joint replacement applications	In vitro, in vivo	Reduced accumulation of metal debris in tissues, decreased risk of inflammation and hypersensitivity	Improved long‐term safety and performance in joint replacement applications	Balancing alloy composition for optimal biocompatibility and mechanical strength remains a challenge	[436, 437, 438, 439]
Doping and coating	Doping and coating Ti implants	Using bioactive ions such as cobalt, Zn, and Mg to tune in vivo immune tolerance of biomaterials, modulating the immune response and reducing inflammation by supporting M2 polarization	In vivo, in vitro	Enhanced M2 polarization	Enhanced biocompatibility, can be designed for specific targeting	Complex fabrication techniques, biodegradation variability	[440, 532, 533]
Polymers	PEG, zwitterionic polymers	localizing release of bioactive factors and providing a matrix for drug delivery (PEG), reduce fibroblast adhesion and decrease the foreign body response (zwitterionic polymers)	In vivo, in vitro	Reduced immune system uptake, prolonged circulation time, induced specific immune responses and tissue integration	Non‐cytotoxicity of degradation products of PEG‐based hydrogels, non‐immunogenic, good biocompatibility and hydrophilicity, ease of surgical insertion	Stability and delivery challenges, potential long‐term effects, limited clinical data, challenges in translating preclinical findings to human application	[444, 445, 446, 447, 449]
C. Biological strategies							
Cell‐based	Stem cells (SCs)	MSCs differentiate into various cell types involved in immune responses, secrete immunomodulatory factors that shift from a pro‐inflammatory to an anti‐inflammatory cell phenotype, suppress T cell activation and proliferation, induce regulatory T cells, inhibit DC maturation and function, reduce pro‐inflammatory cytokines, and secreting appropriate growth factors and ECM proteins to stimulate regeneration and angiogenesis	In vitro, in vivo	Shift from pro‐inflammatory to anti‐inflammatory cell phenotypes, Suppression of T cell activation and proliferation Induction of regulatory T cells, Inhibition of DC maturation and function, Reduced production of pro‐inflammatory cytokines, Stimulated angiogenesis for improved regeneration and healing	Potential for broad application in tissue engineering and regenerative medicine due to their ability to modulate immune responses and promote tissue regeneration	Challenges include standardization of cell isolation, culture, and expansion processes, as well as concerns about long‐term safety and efficacy	[258, 450, 451, 452, 453, 454, 455, 456, 457, 458, 459]
	Preconditioned/modified SCs	Preconditioning the stem cells with different inflammatory agents, genetic manipulation of stem cells, modification of their culture conditions	In vitro, in vivo	Enhanced stem cells’ efficacy and immunomodulation properties	Improved therapeutic potentials of SCs	Donor‐dependent variation	[462]
	Other cell types	– hAMs: Secretion of anti‐inflammatory cytokines and growth factors, inhibition of M1 MPs and neutrophils, promotion of re‐epithelialization, collagen deposition, and angiogenesis – GCsMs: Induction of resolution of inflammation, suppression of T cell proliferation and cytokine release, induction of regulatory T cells and tolerogenic DCs, production of anti‐inflammatory mediators – Modified muscle cells: Enhancement of MP recruitment and polarization towards M2 phenotype, promotion of myogenesis and angiogenesis	In vitro, in vivo	– Improved healing and reduced inflammation in skin wounds (hAM) – Resolution of inflammation and potential treatment of inflammatory diseases (GCsMs) – Enhanced myogenesis and angiogenesis in muscle injury (modified muscle cells)	Potential for targeted and effective immunomodulation, promotion of tissue regeneration, and application across a range of inflammatory and regenerative contexts	Challenges include standardization of cell isolation, modification, and application, long‐term safety and efficacy, and potential for off‐target effects	[380, 463, 470]
Extracellular vesicles (EVs)	MSC‐derived EVs	Activate polarization, cytokine production, antigen presentation, transfer growth factors, anti‐inflammatory cytokines, miRNAs, and mitochondria	In vitro, in vivo, clinical trials	Induced differentiation into anti‐inflammatory phenotypes, participated in inflammatory and immune response regulation	Natural mode of cell communication, potential for targeted delivery	Isolation and characterization challenges, ensuring reproducibility and consistency of content	[80, 81, 82, 83, 464]
Cell membranes	Cell‐derived membranes	Suppressing inflammatory cytokines and promoting anti‐inflammatory mediators, inducing M2 polarization	In vitro, in vivo	Attenuated the immune response, improvement in biocompatibility	Facilitate interactions with surrounding cells, promoting tissue integration and healing, reducing the risk of rejection or adverse reactions	Isolating and maintaining challenges due to their delicate nature, short half‐time	[83, 84, 85]
D. Combination of strategies							
		Combined and synergistic effects	In vitro, in vivo	Enhanced tissue integration, accelerated regeneration, attenuated inflammation, and fibrosis	Integrated approach for comprehensive effects	Complexity in design and implementation, requires extensive optimization	[54, 86, 87, 88]

#### 3.3.1 Physical Strategies

**Figure 11:**
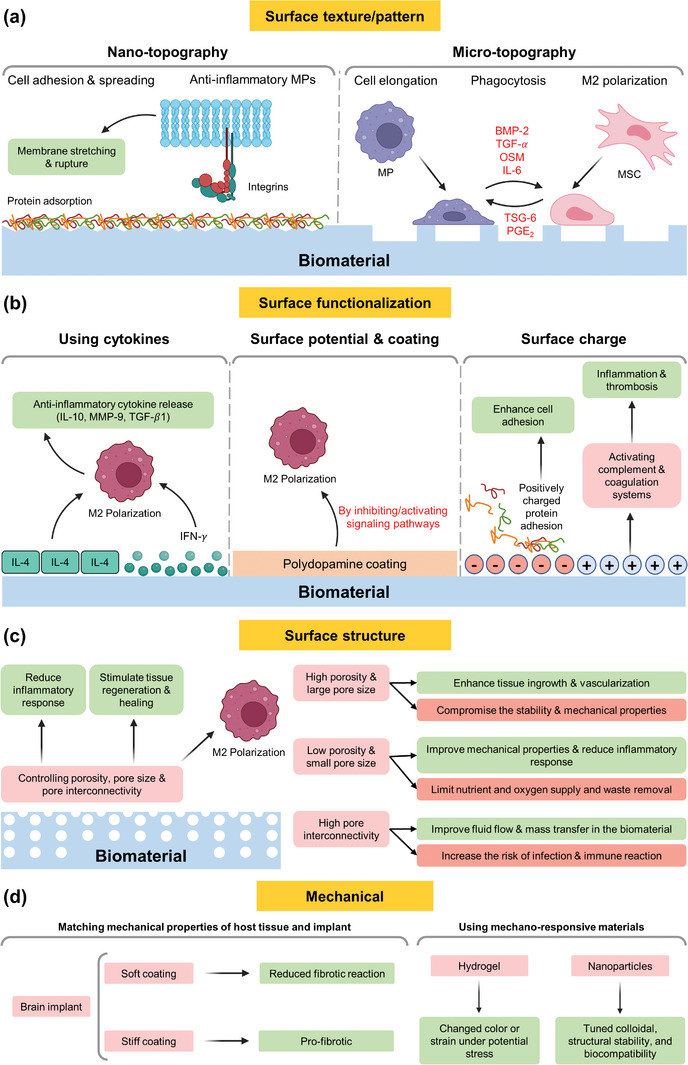
Physical strategies to achieve immunomodulation and engineering of implantable biomaterials using: a) biomaterial surface topography and texture to influence immune cell adhesion and polarization (see also^[^ ^55^ ^]^); b) surface functionalization to modulate immune cells using different methods including cytokines such as Interleukin‐6 (IL‐6) and interferon gamma (IFN‐γ), surface potential, coating, and charge; c) controlled porosity, pore size, and pore interconnectivity for reducing the inflammatory response; d) matching mechanical properties of implanted biomaterial with host tissue and using mechano‐responsive biomaterials. Created with BioRender.com.

Physical strategies comprise shape,^[^ ^276^, ^277^, ^278^ ^]^ surface properties,^[^ ^51^, ^279^ ^]^ structure, porosity, pore size, and pore‐interconnectivity (**Figure** **11**).^[^ ^280^, ^281^, ^282^, ^283^, ^284^, ^285^, ^286^, ^287^, ^288^, ^289^ ^]^ Hydrophilicity, pH, optogenetic,^[^ ^290^, ^291^, ^292^, ^293^ ^]^ electrical,^[^ ^294^, ^295^, ^296^, ^297^, ^298^, ^299^, ^300^, ^301^ ^]^ magnetic,^[^ ^220^, ^302^, ^303^, ^304^ ^]^ magnetoelectric^[^ ^305^, ^306^, ^307^, ^308^ ^]^ properties of the material can also be manipulated and used to control immune response. In addition, mechanical properties also play an important role in relation to the resulting tissue reactions.^[^ ^65^, ^309^ ^]^ For example, surface patterning,^[^ ^53^, ^309^, ^310^, ^311^, ^312^, ^313^, ^314^ ^]^ functionalization,^[^ ^315^, ^316^, ^317^ ^]^ surface potential,^[^ ^84^, ^318^, ^319^, ^320^, ^321^ ^]^ charge,^[^ ^52^ **^,^** ^322^ ^]^ enables M2 polarization by modulating their adhesion, morphology, and signaling pathways, resulting in enhanced tissue regeneration. Similarly, controlling porosity, pore size, and pore interconnectivity enables M2 polarization,^[^ ^323^ ^]^ tissue regeneration,^[^ ^324^ ^]^ and vascularization.^[^ ^324^ ^]^ Biomaterial mechanical properties can be used for immunoengineering, e.g. by using specific mechano‐stimulation^[^ ^325^ ^]^ employing mechano‐responsive materials.^[^ ^325^, ^326^, ^327^, ^328^, ^329^, ^330^, ^331^ ^]^ Implants employing various physical strategies are already in the clinic, e.g. implants that employ mechanical properties matching those of the host target tissue for immune response modulation.^[^ ^55^, ^69^, ^332^, ^333^, ^334^, ^335^, ^336^ ^]^

#### 3.3.2 Chemical Strategies

**Figure 12:**
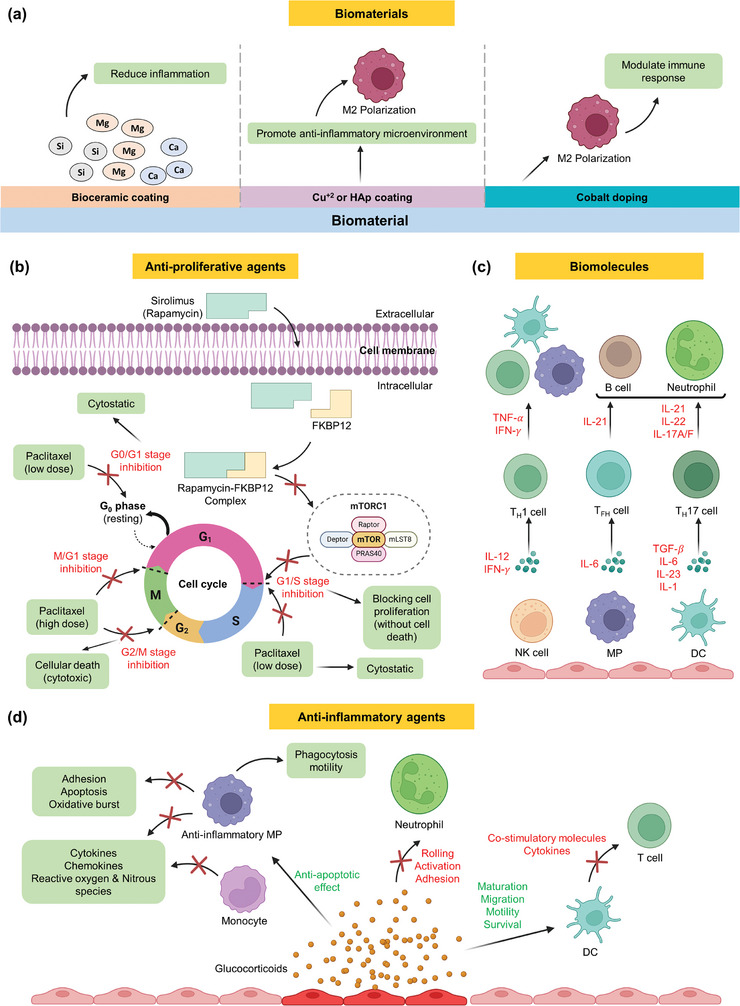
Chemical strategies to achieve immunomodulation and engineering of implantable biomaterials using: a) biomaterials such as bioceramics to modulate the immune response; b) anti‐proliferative agents; c) biomolecules such as cytokines; or d) anti‐inflammatory agents. Created with BioRender.com.

Chemical strategies include interference with events taking place during or after wound healing, such as MP stimulation and polarization (**Figure** **12**).^[^ ^337^, ^338^, ^339^, ^340^ ^]^ We classify them here according to their type, explaining the stage of the immune response they influence. Chemical strategies include the use of pharmaceutical agents including anti‐inflammatory agents (such as steroids,^[^ ^341^, ^342^, ^343^, ^344^, ^345^ ^]^ NSAIDs,^[^ ^34^, ^346^, ^347^, ^348^, ^349^, ^350^, ^351^, ^352^, ^353^, ^354^, ^355^ ^]^ and other agents),^[^ ^356^, ^357^, ^358^, ^359^ ^]^ and antiproliferative agents,^[^ ^360^, ^361^, ^362^, ^363^, ^364^, ^365^, ^366^, ^367^, ^368^ ^]^ biomolecules including peptides,^[^ ^44^, ^226^, ^369^, ^370^, ^371^, ^372^, ^373^, ^374^, ^375^, ^376^ ^]^ cytokines,^[^ ^10^, ^377^, ^378^, ^379^, ^380^, ^381^, ^382^ ^]^ other molecules including O_2_^[^ ^383^, ^384^, ^385^, ^386^ ^]^ and NO^[^ ^387^, ^388^, ^389^ ^]^ or biomaterials such as ECM,^[^ ^390^, ^391^, ^392^, ^393^, ^394^ ^]^ and polymers^[^ ^391^, ^395^ ^]^ to facilitate remodeling, bioceramics with certain compositions to enhance M2 polarization or polymers to enhance regeneration.^[^ ^396^, ^397^, ^398^, ^399^, ^400^, ^401^, ^402^ ^]^ Implants employing chemical strategies for immunomodulation are in clinical trials, such as steroid,^[^ ^403^ ^]^ NSAID,^[^ ^404^, ^405^, ^406^, ^407^ ^]^ and antiproliferative drug^[^ ^408^, ^409^, ^410^, ^411^, ^412^, ^413^, ^414^ ^]^ releasing implants.

#### 3.3.3 Biological Strategies

**Figure 13:**
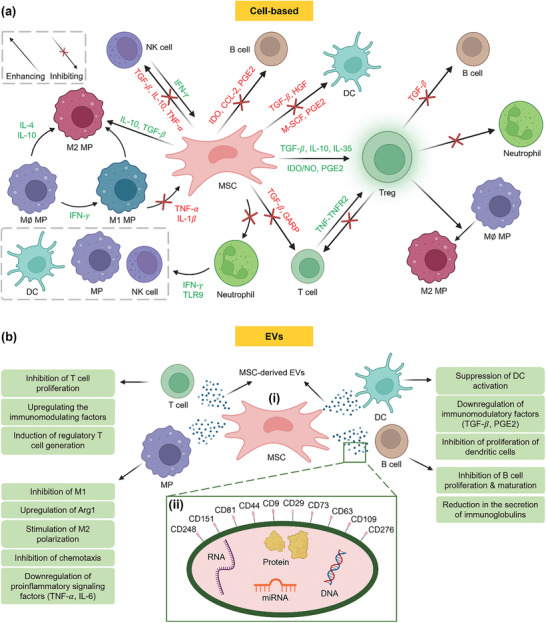
Biological strategies for achieving immunomodulation and engineering. It includes cells and extracellular vesicles (EVs). Cells include stem cells such as mesenchymal stem cells (MSCs). a) MSCs exhibit immunosuppressive effects on macrophages (MPs), T and B cells, regulatory T cells (Tregs), dendritic cells (DCs), neutrophils, and natural killer (NK) cells through the secretion of various cytokines. MSCs could inhibit MP migration and promote a shift from M1 to M2 polarization.^[^ ^468^ ^]^ Created with BioRender.com. b) Illustration showing EVs and their immunomodulatory effects on various cells (i) and molecular content (ii). EVs can induce different immunosuppressive effects and contribute to immunological tolerance (i).  EVs have specific membrane markers and contain various proteins, DNAs, RNAs, and mRNAs (ii). Created with BioRender.com.

Biological strategies involve the use of living cells (such as stem cells,^[^ ^258^, ^415^, ^416^, ^417^, ^418^, ^419^, ^420^, ^421^, ^422^, ^423^, ^424^, ^425^, ^426^, ^427^, ^428^, ^429^, ^430^, ^431^, ^432^, ^433^, ^434^, ^435^, ^436^, ^437^, ^438^, ^439^, ^440^, ^441^, ^442^, ^443^, ^444^, ^445^, ^446^, ^447^, ^448^, ^449^, ^450^, ^451^, ^452^, ^453^, ^454^, ^455^, ^456^, ^457^, ^458^, ^459^, ^460^, ^461^, ^462^, ^463^ ^]^ MPs, fibroblasts, and endothelial cells), EVs,^[^ ^80^, ^81^, ^82^, ^83^ ^]^ and cell‐derived membranes^[^ ^84^, ^85^ ^]^ to guide immune cell behavior and elicit a favorable immune response (**Figure** **13**). These strategies are designed to direct the immune response in a way that supports tissue repair, regeneration, and the resolution of inflammation. Implants that employ biological strategies are already in clinical trials, e.g., using stem cells and mesenchymal stem cell (MSC)‐derived EVs.^[^ ^464^, ^465^, ^466^ ^]^ Strategies employing cells, e.g. stem cell‐based therapies, in particular, require rigorous investigation to ascertain long‐term safety and effectiveness, before they are approved for clinical use.^[^ ^467^ ^]^

#### 3.3.4 Combined Strategies

Combined strategies integrate various approaches from physical, chemical, and biological categories to synergistically enhance immunomodulation. These strategies include, e.g. the co‐delivery of small molecule inhibitors with cells,^[^ ^86^ ^]^ the incorporation of physical cues with cell‐based therapies,^[^ ^87^ ^]^ or the creation of materials that combine multiple functional properties, such as surface texture with controlled release of bioactive molecules.^[^ ^88^ ^]^ The primary goal of combined strategies is to combine the advantages of individual approaches.^[^ ^54^ ^]^ For example, combining physical cues with cell‐based therapy can accelerate regeneration, while integrating surface texture and NO release can enhance the anti‐inflammatory properties of the biomaterial.^[^ ^87^ ^]^ This multi‐modal approach is already being tested in animals to evaluate its effectiveness.^[^ ^86^ ^]^

### 3.4 Modes of Delivery of Immunomodulators

**Figure 14:**
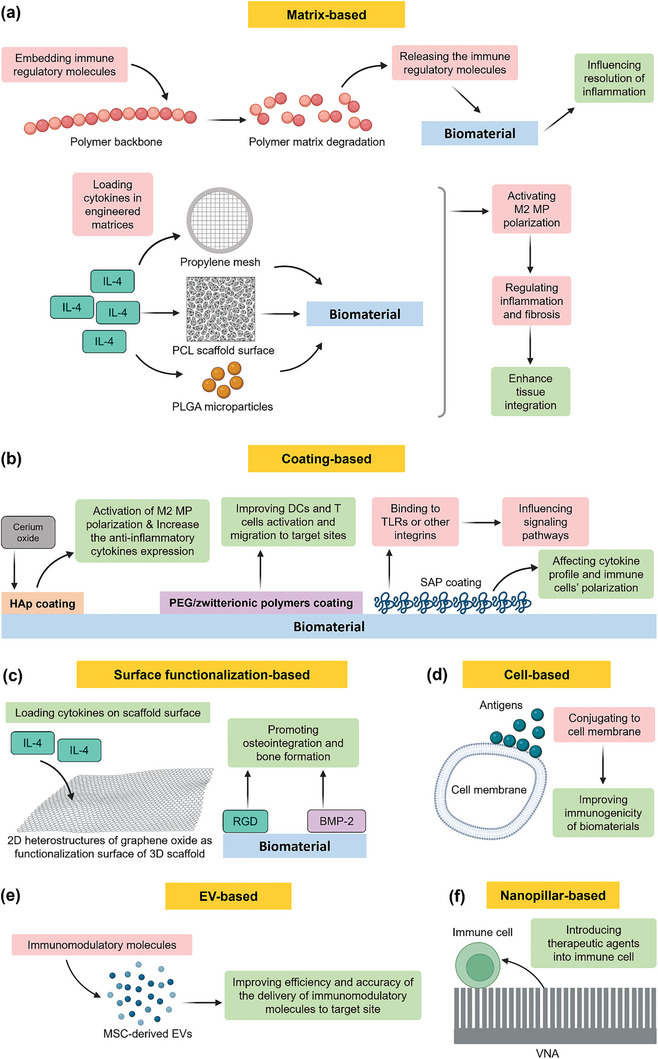
Schematic showing different modes of delivery of immunomodulators: a) Matrix ‐based delivery including embedding the immune regulatory factor/molecules in the polymer backbone in order to become degraded in a well‐conditioned environment and release the immune regulatory molecules to biomaterial surface. This will influence the resolution of inflammation at the target site. A second example of using matrix‐based delivery method is loading the Interloukin‐4 (IL‐4) in engineered matrices such as propylene mesh, polycaprolactone (PCL) scaffold surface, or poly(lactic‐co‐glycolic acid) (PLGA) microparticles to be released at the biomaterial site. The outcome of this transfer would be the activation of M2 macrophage (MP) polarization which will regulate inflammation and then resulting better tissue integration. b) Coating‐based is another method of delivery which is summarized in using specific materials coated on the biomaterial surface in order to modulate the immune response. Hydroxyapatite (Hap) is an example of these materials which incorporate with the cerium oxide and will activate the M2 MP polarization and increase the expression of anti‐inflammatory cytokines. Other examples of coated materials can be polyethylene glycol (PEG) and zwitterionic polymers; these materials can positively affect dendritic cells (DC) T cells activation and their migration to the target sites. Self‐assembling peptide (SAP) is another alternative coating material that binds to toll‐like receptors (TLRs) or integrins and influence the signaling pathways. SAPs also affect cytokines profile and the polarization of immune cells. The third method of delivery is c) surface functionalization‐based delivery. This may include loading interleukin‐4 (IL‐4) on 2D heterostructure of graphene oxide on the surface of 3D scaffold, or functionalizing the surface by Arginylglycylaspartic acid (RGD) and bone morphogenetic protein‐2 (BMP‐2) which will improve osteointegration and bone formation. d) Cell‐based delivery can be explained by an example of antigen conjugation to cell membrane. This incorporation improves immunogenicity of biomaterial. e) Mesenchymal stem cells‐derived extracellular vesicles (MSC‐derived EVs) are a famous example of using EVs for delivering the immunomodulatory molecules. These molecules can be cytokines, chemokines, peptides, or nucleic acids that are loaded into or on the EVs and improves efficiency and accuracy of the immunomodulators’ delivery. f) Nanopillar‐based is a delivery method in which the immune cell can be subjected to therapeutic agents coming from vertical nanowire array (VNA). Created with BioRender.com.

Delivery of immunomodulatory agents and materials can be achieved via matrix‐, coating, surface functionalization‐, cell‐, EV‐, or nanopillar‐based methods (**Figure** **14**). In addition, their release can be controlled by certain triggers, such as changes in the pH or temperature, or by using light or enzymes.^[^ ^73^ ^]^

#### 3.4.1 Matrix‐Based Delivery

Encapsulation of immunomodulatory agents within biomaterials can provide sustained and controlled release, as well as protection from degradation and washout. IL‐4 loaded in charged propylene meshes,^[^ ^469^, ^470^, ^471^, ^472^ ^]^ PLGA microparticles,^[^ ^473^ ^]^ or polycaprolactone (PCL) surfaces^[^ ^474^ ^]^ can lead to M2 polarization and prevention of inflammation and fibrosis, leading to better tissue integration. TGF‐β can also be loaded in implant matrix used to enhance M2 polarization and the differentiation of MSCs.^[^ ^475^ ^]^ Similarly, microspheres have been used to encapsulate antigens and adjuvants to enhance the immune response against pathogens or tumors.^[^ ^476^ ^]^ In another study, EVs derived from human umbilical cord blood mononuclear cells were loaded in silk fibroin scaffolds and led to enhanced anti‐inflammatory cytokine expression and wound closure in diabetic mice skin wounds.^[^ ^477^ ^]^ Another way to modulate the immune response is to incorporate immune regulatory molecules in the polymer backbone, allowing them to be released from the matrix under specific conditions. For instance, embedding the immune regulatory molecule, Itaconate (ITA), in a polymer backbone enables its release through hydrolytic degradation of the polymer matrix, circumventing the problem of its delivery.^[^ ^478^ ^]^

#### 3.4.2 Coating‐Based Delivery

Coatings such as peptides,^[^ ^479^ ^]^ polymers,^[^ ^443^ ^]^ or nanoparticles^[^ ^480^ ^]^ can be used for the delivery of immunomodulatory strategy/agent. Peptides such as SAPs can also be used for drug delivery, or vaccine development. SAPs can also modulate the immune system by binding to specific receptors on immune cells, such as TLRs or integrins, and activating or inhibiting signaling pathways that regulate immune cell function.^[^ ^225^, ^226^ ^]^

#### 3.4.3 Surface Functionalization‐Based Delivery

Studies suggest that functionalization approach can employ different peptides, cytokines and immune regulatory drugs. In addition, functionalization enables controlled delivery of the immune‐responsive drugs at the implantation site. This can address the challenges of delivering growth factors such as TGF‐β1 and endow immunoregulatory properties to the biomaterials. Zwitterionic polymers have been used to conjugate chemokines such as stromal cell‐derived factor‐1 (SDF‐1) and C‐C motif chemokine ligand 21 (CCL21) to recruit endothelial progenitor cells (EPCs) and DCs to the implant. Similarly, Ti implants have been modified with peptides such as RGD and bone morphogenetic protein‐2 (BMP‐2) to promote osteointegration and bone formation. On demand delivery of IL‐4 via functionalization of a biomaterial surface by immobilizing of IL‐4/polydopamine (PDA) coating on a black TiO_2_ nanotube (B‐TNT) surface can be achieved using near infrared (NIR) irradiation.^[^ ^481^ ^]^ Similarly, IL‐4 can also be loaded on 3D scaffold functionalized with 2D heterostructures of black phosphorus and graphene oxide.^[^ ^482^ ^]^ The overall assembly of the scaffold was found to facilitate the release of IL‐4 from the scaffold, thereby promoting osteogenesis and angiogenesis by supporting a pro‐healing microenvironment in the bone defect.^[^ ^483^ ^]^ Anti‐inflammatory peptides, such as alpha‐MSH tridecapeptide, can also be tethered to biomaterial surfaces to create an immunosuppressive microenvironment. For example, Alpha‐MSH tridecapeptide coating of silicon‐based cerebral implants can limit the inflammatory response and enhance implant stability.^[^ ^484^, ^485^ ^]^

#### 3.4.4 Cell‐Based Active Agent Delivery

These delivery systems^[^ ^486^ ^]^ offer advantages such as prolonged release, targeting to specific cell compartments, and biocompatibility. These systems can use cells themselves,^[^ ^487^ ^]^ cell membranes,^[^ ^84^ ^]^ or synthetic cells.^[^ ^488^, ^489^, ^490^ ^]^ These cells can be of plant,^[^ ^491^ ^]^ bacterial,^[^ ^492^, ^493^ ^]^ animal,^[^ ^494^ ^]^ or human^[^ ^495^ ^]^ origin. Bacterial cells have also been used as non‐living carriers, by removing the cytoplasmic content and retaining the morphology and antigenic structures.^[^ ^493^ ^]^ Because of their potential to serve as carriers for various protein drugs and conventional pharmaceutical products, and macroparticles, RBCs have been investigated.^[^ ^496^ ^]^ Cargoes such as cytokines, chemokines, antigens, and drugs have been used for modifying implant design and properties, and included in cells as encapsulated or conjugated molecules using various methods such as electroporation, osmotic shock, or chemical cross‐linking.^[^ ^497^ ^]^ For example, cytokines such as IL‐4 and IL‐10 have been encapsulated in RBCs for immunomodulation.^[^ ^498^ ^]^ Antigens have also been conjugated to cell membranes to enhance the immunogenicity of biomaterials.^[^ ^499^, ^500^ ^]^ Drugs such as dexamethasone have been loaded in macroparticles to reduce the fibrotic reaction to biomaterials.^[^ ^501^ ^]^

#### 3.4.5 Extracellular Vesicle (EV)‐Based Delivery

Engineered EVs can be used as delivery systems of desired molecules or substances.^[^ ^83^ ^]^ They can safely travel in extracellular fluids and taken up by cells. They can enhance the efficiency of the delivery of immunomodulatory factors/molecules such as cytokines, chemokines, peptides, or nucleic acids that can be loaded into the EVs^[^ ^502^ ^]^ or on their surface^[^ ^82^ ^]^. Multimodal engineering enhances the targeting precision and therapeutic efficacy of EVs.^[^ ^503^ ^]^ For instance, research has highlighted the delivery of clustered regularly interspaced short palindromic repeats (CRISPR)‐associated 9 (Cas9) protein and subgenomic RNA (sgRNA) using EVs for gene editing.^[^ ^504^ ^]^ This approach integrates genetic engineering to express Cas9 protein on the surface of EVs, chemical modification linking sgRNA to Cas9 protein via click chemistry, physical method of electroporation to boost loading efficiency, and bio‐orthogonal chemistry for EV labeling with fluorescent probes to enable tracking.

#### 3.4.6 Nanopillar‐Based Delivery

The emerging field of programmable nano‐biointerfaces facilitates biomolecular delivery,^[^ ^505^ ^]^ immunomodulation,^[^ ^506^ ^]^ and immunotherapy.^[^ ^507^ ^]^ Recent advances have shown that vertical nanostructure array‐mediated intracellular delivery can be a promising platform for cell‐based immunotherapy, as it can be used to introduce exogenous genetic and therapeutic agents into immune cells, allowing rapid and direct intracellular access with minimal cytotoxicity.^[^ ^508^ ^]^

## 4 Current Challenges and Future Perspectives

**Figure 15:**
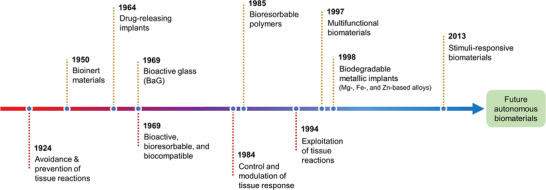
Illustration showing milestones in the evolution of ideas to address tissue reactions to biomaterials, strategies, from past to present and future. These milestones comprise bioinert materials,^[^ ^509^ ^]^ drug‐releasing implants,^[^ ^510^ ^]^ bioactive glass (BaG),^[^ ^511^ ^]^ bioresorbable polymers,^[^ ^512^, ^513^, ^514^ ^]^ multifunctional biomaterials,^[^ ^515^, ^516^ ^]^ biodegradable metallic implants,^[^ ^517^, ^518^, ^519^ ^]^ and stimuli‐responsive materials.^[^ ^520^ ^]^ The representation also shows the objectives in developing future autonomous biomaterials. Objectives of research first focused on the prevention of tissue reactions.^[^ ^521^ ^]^ Then, the focus was put on leveraging tissue reactions to useful applications,^[^ ^522^ ^]^ and modulating tissue response,^[^ ^523^, ^524^ ^]^ leading to the development of automated implants in the future.

With increased understanding of immune reaction on molecular and cellular levels, new tools and approaches became available, which do not only enable the control of immune responses but also engineering them. In the future, it is expected that implants will be self‐aware, self‐healing, and immunomodulating, enabling better implant function and durability (**Figure** **15**). However, there are still challenges on the way to achieving this as well as potential solutions that need to be explored further.

### 4.1 Challenges

Because the immune system is complex, dynamic, and heterogeneous, identifying targets for immunomodulation and engineering is a major challenge in this field. Immune responses also depend on various properties of the biomaterial. Therefore, identifying targets requires a comprehensive and multidisciplinary study that can integrate various tools and approaches used in disciplines such as immunology, bioengineering, materials science, nanotechnology, and bioinformatics. Different applications may have different goals and requirements of immune reaction modulation. Therefore, developing standardized methods and criteria for evaluating immunoengineering is essential for advancing the field and facilitating the translation of biomaterials into clinical practice.

Agents used for immunomodulation may have different effects on different targets, depending on the dose, duration, frequency, and combination. Moreover, such agents may also have unwanted effects. Therefore, their delivery requires a rational design. For example, anti‐inflammatory drugs can inhibit bone formation and impair implant integration.^[^ ^525^, ^526^ ^]^ The dose of the agents or strategies is important for achieving the desired therapeutic effect. Too high or too low dose may cause toxicity or inefficacy, respectively. The duration of the release should be controlled to match the healing time of the tissue or organ. The combination of different agents or strategies may create synergistic or antagonistic effects that can enhance or reduce their therapeutic efficacy.^[^ ^527^ ^]^ Therefore, the compatibility and stability of different agents should be carefully controlled. There are also some challenges and limitations associated with biomaterials used for their delivery, including the complexity and variability of the immune system, as well as the lack of standardized methods and criteria for evaluating parameters, and safety issues. The carrier material should ideally be biodegradable and responsive to stimuli, such as changes in pH, temperature, or light, to allow for temporal regulation of the release.

Engineering of the targeting mechanisms is another challenge. For instance, stimuli and agents that are delivered by biomaterials may have different effects on different targets. Therefore, targeting requires careful design, such as the use of targeting ligands, to enhance the selectivity and specificity of targeting mechanisms. For example, antibodies can be used to target specific receptors or cytokines on the surface of innate immune cells, such as MPs, neutrophils, or natural killer cells, and modulate their activation or inhibition. Another factor that affects targeting is the dynamic nature of the immune system and local microenvironment. For example, a biomaterial that induces an anti‐inflammatory response in one condition may induce a pro‐inflammatory response in another condition.^[^ ^91^ ^]^ Therefore, developing biomaterials that can sense and adapt to the changing microenvironment and immune system is a challenge that requires smart design.

The specificity and selectivity of biomaterials for certain immune cell subsets or tissues also influence the targeting process. For example, Tregs are critical for suppressing autoimmunity and inflammation. Therefore, targeting Tregs with biomaterials may be beneficial for treating autoimmune diseases or enhancing tissue regeneration. However, targeting Tregs may also have negative consequences for other aspects of immunity, such as anti‐tumor or anti‐infection responses.^[^ ^528^ ^]^ Therefore, designing biomaterials that can target specific immune cell subsets without affecting other cells or organs is a challenge that requires precise and accurate targeting mechanisms. The immune system and its modulation are influenced by various factors that affect the targeting of therapeutic agents. For example, combining different types of immunomodulatory factors or cells into biomaterials may enhance or complement their effects. However, combining too many factors or cell types may also cause interference or imbalance in the immune system. Therefore, designing biomaterials that can modulate the immune system in a complex and diverse manner is a challenge that requires rational and integrative design.

The stability of biomaterials and their cargo during storage, transportation, and administration represents another challenge. It can be affected by factors such as temperature, humidity, light, oxygen, pH, enzymes, and contamination. To overcome this, strategies such as adding stabilizers, preservatives, antioxidants, or antimicrobials can be employed. Novel biomaterials and their cargo that can be stable or resistant to degradation or contamination should also be developed. Another challenge related to delivery is to ensure the specificity of the biomaterials and their cargo to the target immune cells, tissues, or organs. Specificity can be influenced by factors such as size, shape, surface charge, hydrophobicity, affinity, or ligand‐receptor interactions. For example, some biomaterials and their cargo can be rapidly cleared by the reticuloendothelial system (RES) or renal filtration due to their large size or negative charge. Some biomaterials or cargos can cause nonspecific binding or uptake by non‐target cells or tissues due to their high hydrophobicity or affinity. They can induce unwanted immune responses or side effects due to their foreignness or toxicity. To overcome this, biomaterials and cargos can be functionalized with cell‐specific molecules, including antibodies or receptor‐targeting peptides. These modifications enhance the biomaterial's surface interaction with cell membranes, improving both affinity and specificity.^[^ ^529^, ^530^ ^]^ They can also be designed to respond to specific stimuli that trigger the activation of the biomaterial or the release of cargo at the target site.^[^ ^531^, ^532^, ^533^ ^]^

Immunoengineering may have unintended or unwanted effects. For example, biomaterials may induce an excessive or inappropriate immune response that causes inflammation, infection, allergy, or autoimmune diseases. Moreover, developed immunomodulation methods may interfere with systems such as homeostasis of the host and the microbiome, which may impact the health of the host. Therefore, ensuring the safety and biocompatibility of immune modulating systems is crucial for preventing or minimizing the potential adverse effects and risks. In addition, immunoengineering may raise ethical and regulatory issues that need to be addressed. For example, biomaterials may involve the use or manipulation of living organisms or their products, such as cells, EVs, genes, or proteins. Therefore, respecting ethical and regulatory principles and guidelines for immunoengineering is important for ensuring the protection of the rights and interests of patients.

One of the major challenges in engineering immune response to biomaterials is the difference between in vitro and in vivo observations. For example, MP polarization is often used as a readout of the immunomodulatory properties of biomaterials in vitro, but it may not reflect the complex and dynamic interactions between biomaterials and immune cells in vivo. Because different biomaterials induce different immune responses, there is a need for more standardized and in vivo representative in vitro models and procedures that accommodate recent advances in understanding immune responses and that can be more comprehensive, enough to understand and predict immune response that may take place in vivo. There are also some challenges and limitations associated with immune cell types used such as the difficulty of obtaining sufficient numbers of cells from donors or patients, allergenicity and risk of infection, loss of transplanted cells, as well as the ethical and regulatory implications of manipulating them. Unlike stem cells, immune cells have limited self‐renewal potential, and may require additional modifications or support to enhance their function and survival in vivo.^[^ ^534^, ^535^ ^]^ There are also challenges associated with the use of EVs for the immunomodulation, such as difficulties associated with their isolation, delivery and preservation at the target location.

Another challenge is the regulatory constraints facing multifunctional biomaterials that aim to engineer immune tolerance. For example, drug releasing and cell containing biomaterials have more complicated path to approval. Antigen‐specific and nonspecific tolerogenic agents, such as anti‐inflammatory drugs, and biomolecules can be useful for modulating tissue response. However, they add to complexity and extension of regulatory approval. Similarly, the use of tolerogenic cells, such as regulatory T cells or DCs represents a challenge and adds complexity. Therefore, these multifunctional biomaterials pose several regulatory challenges. In addition, challenges include ensuring the safety, quality, and consistency of the biomaterial components, demonstrating the efficacy and durability of the tolerogenic effect, and addressing the ethical issues related to the manipulation of the immune system.

### 4.2 Future Perspectives

The direction of research in immunomodulation and engineering points to the development of more precise, personalized, and effective strategies that can target specific immune cells, molecules, or pathways involved in disease pathogenesis and regeneration. For example, some of the current approaches include the use of monoclonal antibodies,^[^ ^536^, ^537^ ^]^ cytokines,^[^ ^536^ ^]^ vaccines,^[^ ^538^ ^]^ gene therapy,^[^ ^539^ ^]^ cell therapy,^[^ ^540^, ^541^ ^]^ biomaterials,^[^ ^67^ ^]^ and nanotechnology.^[^ ^542^ ^]^ Furthermore, targeting biomaterials can modulate not only the innate and adaptive immune systems, but also the neuroendocrine system and metabolism, which are intimately connected. Carefully engineered, strategies of immune engineering can improve diagnosis, prevention, and treatment of various immune‐related diseases.

To improve the methods of immunomodulation, future research needs to focus on developing more integrated and multimodal strategies that can leverage the benefits of different methods. These may include creating smart biomaterials that can adapt to the local immune environment and change their properties, functionality, or delivery according to need. They can also include designing hybrid biomaterials that combine natural or synthetic immune cells or tissues to form immunomodulatory niches or organs. Developing biomaterials that can customize their immunomodulatory effects based on the individual's immune profile and disease state would also be an important focus of research.

A possible direction for improving delivery is to develop hybrid delivery systems that can combine different types of biomaterials or delivery methods to achieve synergistic effect. These systems can be based on various strategies, such as co‐delivery, sequential delivery, hierarchical delivery, or modular delivery. For example, co‐delivery systems can deliver two or more biomaterials or cargos simultaneously to enhance their immunomodulatory effects. Sequential delivery systems can deliver biomaterials or cargos in a predetermined order to achieve optimal immune responses. Hierarchical delivery systems can deliver biomaterials or cargos in a nested or layered structure to achieve multiple functions.

An important future research direction should be the development of biomimetic or biologically inspired biomaterials that can mimic or emulate the structure, function, and behavior of natural biological systems, such as cells, tissues, and organs. These biomaterials can be based on strategies such as self‐organization,^[^ ^543^ ^]^ self‐healing,^[^ ^89^, ^222^, ^544^ ^]^ self‐regulation,^[^ ^545^ ^]^ and self‐adaptation.^[^ ^546^ ^]^ These bioinspired biomaterials can enable more natural and harmonious integration with the host immune system and the surrounding tissues or organs.^[^ ^152^ ^]^ Another possible direction is to develop bioresponsive biomaterials that can modulate or influence the immune response to certain triggers.^[^ ^67^ ^]^ These biomaterials can be used for immunostimulation, immunosuppression, immunotolerance, and immunoregulation.

There are also possibilities to develop technologies that can influence the stages and control them in terms of events and duration of the FBR. By manipulating temporal and spatial aspects of the stages of FBR using biomaterial design or external stimuli, it may be possible to modulate FBR and achieve a more favorable outcome. Another possibility is to use a combinatorial approach (chemical and physical means) to engineer immune response to biomaterials. By combining these two, a better control of the immune response can be achieved. A third possibility is to develop multimodal devices that use sensors, actuators, and communication systems to monitor and modulate the immune response to biomaterials in real time. This may enable more precise and personalized immunomodulation. A fourth possibility is to use microphysiological systems (MPS) such as organoids,^[^ ^547^, ^548^ ^]^ organ‐on‐a‐chip (OoC) systems,^[^ ^549^, ^550^, ^551^ ^]^ and 3D bioprinted constructs^[^ ^552^ ^]^ to study the immune response to biomaterials. These 3D in vitro models help to circumvent limitations of 2D cell culture methods and the need for the use of experimental animals. By using MPS models that incorporate immune cells,^[^ ^553^ ^]^ it may be possible to better simulate and understand the complex and dynamic interactions between biomaterials and the immune system in vitro. They can also be used to move to clinical trials as they are now defined as one of the non‐clinical methods that can be used for filing with regulatory organizations.^[^ ^554^ ^]^ A fifth possibility is to use AI to analyze and optimize the immune response to biomaterials. AI can be applied to various aspects of engineering immune response to biomaterials, such as designing novel biomaterials with desired immunomodulatory properties, predicting the immunological outcomes of biomaterial implantation, identifying biomarkers or targets for immunomodulation, and personalizing immunotherapy based on individual characteristics. AI can, therefore, accelerate the discovery and development of more effective immunotherapies.

It is envisioned that future implants will be somehow autonomous. They will be able to sense, process, and respond to the physiological signals and conditions of the host or the environment, without the need for external intervention or control.^[^ ^555^ ^]^ Such implants can modulate the immune system and engineer functional tissues or organs by delivering appropriate stimuli to achieve precise and effective modulation of the immune system. To achieve this, the integration of different science and technology disciplines is required, and innovative solutions that take place at the interface of these fields will provide an answer to many open questions in the field of immunoengineering.

## 5 Conclusions

Biomaterials are used to help cells and tissues to perform certain functions such as healing and regeneration, and to induce certain responses such as vaccination against infection or tumors, or tolerance of transplants. Biomaterials have evolved from being bioinert to be bioactive, bioresponsive, and biomimetic smart biomaterials. Research is moving forward by integrating advances made in various fields such as biology, chemistry, physics, engineering, and electronics toward the development of autonomous implants. Methods to prevent, control, modify, and modulate molecular and cellular responses to biomaterials involves various chemical, physical, biological, and combinatorial approaches. Although success in the use of biomaterials as implants in the cardiovascular and skeletal systems has largely been met, there are still challenges facing the control of immune responses by immunoengineering. Future directions in research into the field of immunomodulation warrant the integration of several technologies and disciplines leading to successful clinical translation of immunoengineering, precision, and individualized medicine.

## Conflict of Interest

The authors declare no conflict of interest.
